# Resolving consecutive excited-state evolution in Fe-amido chromophores by wide-band optical transient absorption spectroscopy

**DOI:** 10.1039/d5sc06464c

**Published:** 2026-01-15

**Authors:** Christina Wegeberg, Baldeep K. Sidhu, Pavel Chábera, Jens Uhlig, Rory A. Cowin, Julia A. Weinstein, Petter Persson, Arkady Yartsev, David E. Herbert

**Affiliations:** a Division of Chemical Physics, Department of Chemistry, Lund University 22100 Lund Sweden arkady.yartsev@chemphys.lu.se; b Department of Chemistry and the Manitoba Institute for Materials, University of Manitoba 144 Dysart Road Winnipeg Manitoba R3T 2N2 Canada david.herbert@umanitoba.ca; c Department of Chemistry, University of Sheffield Sheffield S3 7H UK; d Division of Computational Chemistry, Department of Chemistry, Lund University 22100 Lund Sweden petter.persson@compchem.lu.se

## Abstract

A detailed understanding of excited-state evolution is critical to realizing the full potential of abundant-metal coordination complex photosensitizers. Here, we show how wide-band optical transient absorption spectroscopy (oTA) can delineate the complete energy relaxation pathway of the photoexcited state of Fe(ii) polypyridyl complexes supported by benzannulated diarylamido ligands. By covering a broader spectral region from 370 to 1200 nm, we resolve consecutive evolution of a photoexcited Fe-amido chromophore from an initially generated singlet ‘π-antibonding-to-ligand’ charge transfer (^1^PALCT) excited state to a long-lived metal-centred quintet (^5^MC) *via* both a ^3^PALCT and what we assign as a ^3^MC state. Notably, we identify spin-parity transformations by observing photogeneration of the ^1^PALCT followed by its conversion into a ^3^PALCT state, and the subsequent ^3^MC-to-^5^MC transformation *via* observation of an isosbestic point in the oTA spectral dynamics. The state-to-state transformations are accompanied by coherent oscillations which are impulsive Raman-induced, originating in the ground state. Combining high-resolution, wide-band oTA experiments with the unique absorptive properties of diarylamido ligand–metal complexes, we are thus able, for the first time, to trace the complete deactivation trajectory of an iron(ii) polypyridyl sensitizer using optical spectroscopy.

## Abstract 2

Combining high-resolution, wide-band oTA experiments with the unique absorptive properties of diarylamido ligand-metal complexes, we trace the complete deactivation trajectory of an iron(ii) polypyridyl sensitizer using optical spectroscopy.

## Graphical abstract

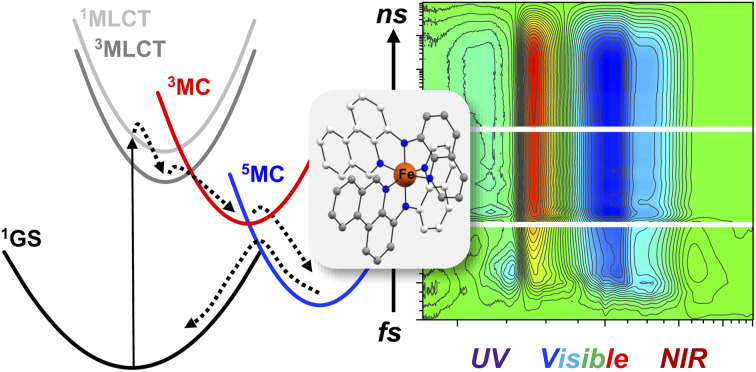

## Introduction

The light-mediated reactivity of transition metal complexes holds tremendous promise in solar energy harvesting,^1,2^ photocatalysis,^3^ biomedical phototherapy,^4,5^ and beyond. While recent reports have shown that locally excited metal-centered (MC) excited states can, in certain instances, be profitably exploited,^6–12^ such chemistry is generally considered most feasible when driven by the chemical potential inherent to charge-separated excited states.^13^ Understanding the character of a molecule's light-generated electronic excited states and their temporal evolution is therefore critical to unlocking the full potential of transition metal-based photosensitizers. For (pseudo-)octahedral, low-spin d^6^ coordination complexes bearing π-conjugated organic ligands, light absorption first generates charge-separated states with ‘metal-to-ligand charge transfer’ (MLCT) character, involving promotion of an electron from the filled metal-based, *t*_2g_-type orbital set into vacant low-lying, ligand-localized orbitals.^14^ For chromophores containing 4d or 5d transition metals, the ligand field strength of these heavier metals ensures that MC states, involving population of the *e*_g_ metal-based manifold, are destabilized relative to such MLCT states. This arrangement typically suppresses significant participation of MC states in deactivating the MLCT excited state, leading to nanosecond (ns) or longer MLCT lifetimes that can enable bimolecular reactivity.^15^ In comparison, for lighter first-row transition elements such as iron, intrinsically weaker ligand fields yield energetically accessible MC states which often facilitate rapid deactivation of the initially photoexcited MLCT state.^16^

**Fig. 1:**
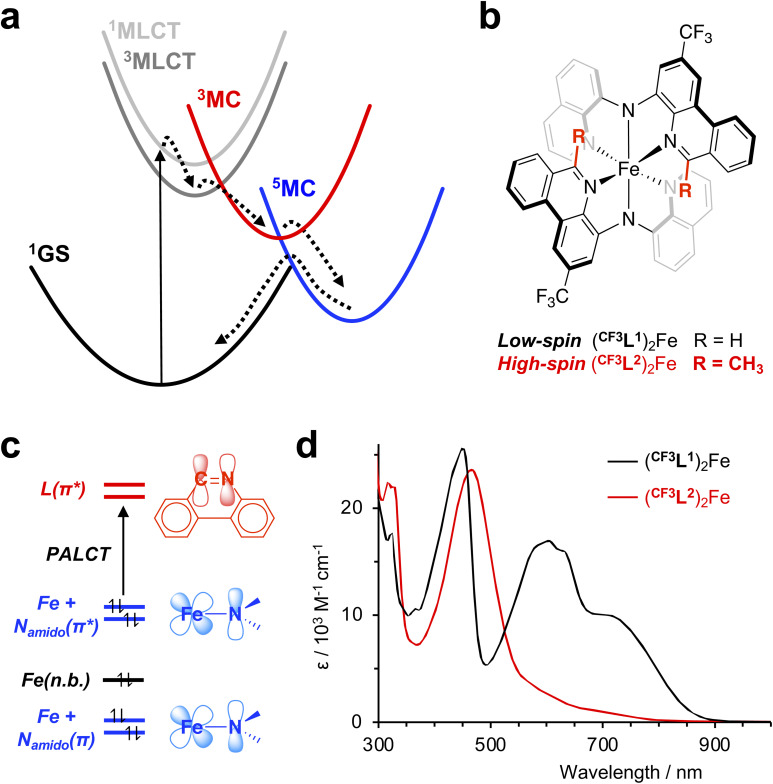
(a) ^1^MLCT → ^3^MLCT → ^3^MC → ^5^MC → ^1^GS decay cascade typical of iron(ii) polypyridyl complexes such as [Fe(bpy)_3_]^2+^; (b) molecular structure of the low-spin iron(ii)-amido chromophore (^CF3^L^1^)_2_Fe and its high-spin analogue (^CF3^L^2^)_2_Fe; (c) simplified molecular orbital (MO) diagram showing the strong Fe(3d)-N_amido_(2p) mixing that leads to ‘π-antibonding-to-ligand’ charge-transfer (PALCT) character to the lowest energy absorption manifold of (^CF3^L^1^)_2_Fe; (d) overlay of the steady state UV-Vis-NIR absorption spectra of (^CF3^L^2^)_2_Fe and (^CF3^L^1^)_2_Fe in toluene at 295 K.

The successful deployment of spectroscopic techniques has been key to understanding these differences in excited-state dynamics. For example, femtosecond (fs) fluorescence spectroscopy was used to directly observe the initially generated ^1^MLCT state of the canonical chromophore ruthenium(ii) tris(2,2′-bipyridine), [Ru(bpy)_3_]^2+^, and determine its lifetime (the most recent estimate being ∼20 fs),^17^ while optical transient absorption (oTA) spectroscopy confirmed formation of a long-lived ^3^MLCT excited state ∼300 fs after photoexcitation.^18^ Similarly, ultrafast studies of [Fe(bpy)_3_]^2+^ helped elucidate differences in the photophysical landscapes of 4d^6^ and 3d^6^ coordination complexes; thus, fs fluorescence upconversion and TA experiments established that the photogenerated ^1^MLCT state of [Fe(bpy)_3_]^2+^ converts to a ^3^MLCT state in ≤20 fs *via* intersystem crossing, followed by swift deactivation by ultrafast population of a quintet metal-centered state (^5^MC).^19^ While a follow-up XANES study^20^ proposed direct population of the quintet state from the charge-transfer manifold (*i.e.*, ^1^MLCT → ^3^MLCT → ^5^MC), modelling of femtosecond iron Kβ X-ray fluorescence spectroscopy data^21,22^ best fit the intermediacy of a triplet metal-centered (^3^MC) excited state. These experiments established the now-accepted ^1^MLCT → ^3^MLCT → ^3^MC → ^5^MC → ^1^GS (GS = ground state) decay cascade for iron(ii) polypyridyl complexes (Fig. 1a), though the precise details remain a matter of contemporary debate.^23^ These various studies highlight how combining sophisticated spectroscopic techniques can be a powerful approach to addressing the challenge of accurately describing a molecule's excited state topography. In principle, oTA itself can unveil a complete sequence of processes including CT-to-MC transitions and sense changes related to transitions between states of different spin parity. The relaxation dynamics of [Fe(bpy)_3_]^2+^, however, do not result in a period in which a majority of electronically excited molecules exist in the ^3^MC configuration. This has hampered identification of the ^3^MC state by optical spectroscopy and contributed to the debate over the exact decay pathway. Here we showcase the capability of oTA by experimentally observing, for the first time, the complete ^1^MLCT → ^3^MLCT → ^3^MC → ^5^MC decay cascade of an iron(ii) polypyridyl, aided by improved time/spectral resolution, the unique absorptive properties of diarylamido ligand complexes, and reference spectra modelling absorption signatures of various excited states.

Specifically, we revisited the oTA spectroscopic characterization of a ferrous compound supported by a benzannulated diarylamido ligand {(^CF3^L^1^)_2_Fe; Fig. 1b}. Such complexes broadly absorb visible light^24^ thanks to highly covalent Fe-N_amido_ bonds^25^ with mixing of filled Fe(3d) and N_amido_(2p) orbitals resulting in highest occupied molecular orbitals (HOMO, HOMO-1) with Fe-N_amido_ π-antibonding overlap. This electronic structure yields vertical excitations with ‘π-antibonding-to-ligand’ charge-transfer (PALCT) character (Fig. 1c).^26^ Low-energy excitation (*λ*_ex_ = 780 nm) generates an excited state with a lifetime on the order of ∼3 ns and strong absorptive properties of its own. Previous oTA experiments covering the UV/visible region of the electromagnetic spectrum (400–800 nm) provided spectroscopic signatures that were assigned to formation of a PALCT excited state based first on comparison with the absorption spectrum of the oxidized complex,^24^ then later bolstered with a simulated TA spectrum obtained using spectroelectrochemically generated spectra of both the oxidized and reduced species.^25^ Re-examination of the UV/Vis oTA data using a synthetic high-spin model complex to mimic the quintet ligand-field excited state, however, showed that the formation of a long-lived ^5^MC excited state was also consistent with the features observed by TA spectroscopy. A subsequent ultrafast X-ray emission spectroscopy (XES) study supported the assignment of the long-lived electronic excited state as a metal-centered quintet spin-state configuration, but did not robustly observe the initially generated ^1^CT excited state or a ^3^MC intermediate.^27^ Here, using ultrafast fs resolution TA spectroscopy with improved signal-to-noise^28^ covering a wider spectral range from the UV to the near infrared (NIR) region, we show definitive discrimination of the PALCT and MC excited states and uncover the complete photophysical trajectory which precedes establishment of the long-lived ^5^MC electronic state in these iron(ii) polypyridyls.

## Results and discussion

We selected the representative complex (^CF3^L^1^)_2_Fe for our study (Fig. 1b). (^CF3^L^1^)_2_Fe is a low-spin (3d^6^, *S* = 0) pseudo-octahedral complex whose preparation and characterization has been previously described.^24^ We also include the 6-methyl-substituted analogue (^CF3^L^2^)_2_Fe with a high-spin quintet ground state, to serve as a synthetic model of the ^5^MC ligand-field excited state of (^CF3^L^1^)_2_Fe (*vide supra*). The synthesis and characterization of (^CF3^L^2^)_2_Fe has also been reported.^27^ At room temperature, the electronic absorption spectra of (^CF3^L^2^)_2_Fe contains intense bands in the UV region and a strong band in the visible region with a maximum at 470 nm (*ε* = 23 600 M^−1^ cm^−1^) and a low-energy tail reaching to ∼750 nm (Fig. 1d). We note little solvatochromism is observed with this class of compounds and the absorption spectrum in lower polarity solvents (*e.g.*, toluene) closely resemble those in more polar solvents (*e.g.*, acetonitrile).^25^ Time-dependent density functional theory (TD-DFT) simulations of the absorption spectrum of (^CF3^L^2^)_2_Fe assigned the bands in the UV region to ligand-localized transitions, while the strong band in the visible region was tracked to transitions with intraligand charge-transfer (ILCT) character and the low energy tail at 500–800 nm is dominated by MLCT transitions.^27^ Despite weaker metal–ligand interactions induced by increased ligand sterics leading to stabilization of the high-spin ground state, the combination of π-donor amido ligands and benzannulated acceptor arms still conspire to produce a rather unique absorption profile for the high-spin (^CF3^L^2^)_2_Fe exemplar; generally, Fe(ii) polypyridyl complexes with high-spin quintet ground states only weakly absorb visible light.^29–31^

A ^1/3^PALCT excited state for (^CF3^L^1^)_2_Fe should, in principle, bear characteristics reminiscent of the optical signatures associated with reduction of a ligand arm and oxidation of the N_amido_-Fe-N_amido_ core which can be modelled using spectroelectrochemistry (see Fig. S1–S4).^32^ The transient absorption spectrum of a ^5^MC excited state, on the other hand, would be expected to more closely resemble the difference spectrum simulated by subtracting the ground state absorption spectrum of the low-spin complex (^CF3^L^1^)_2_Fe from that of the high-spin complex (^CF3^L^2^)_2_Fe. We previously noted qualitative similarities in the visible region^27^ including the positive feature at ∼480 nm and negative bleach between ∼500-800 nm, but expanding the spectral windows shows clear divergence in the UV and NIR, and quantitative discrepancies in the extinction values for features in the visible region (Fig. 3a).

**Fig. 2:**
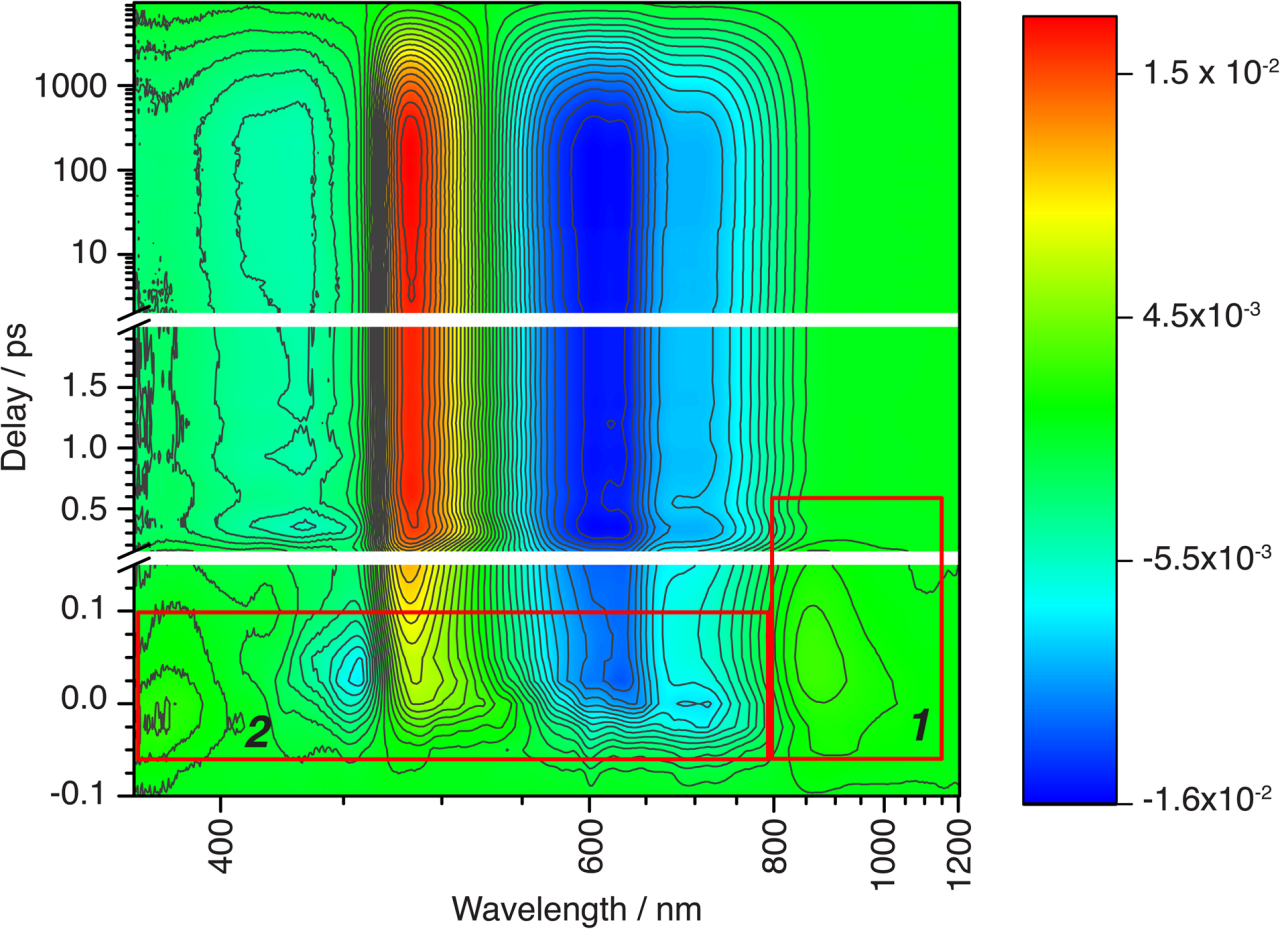
Contour colour-filled plot of chirp-corrected oTA spectral dynamics over the entire covered spectral/temporal range. The red boxes outline the spectral/temporal windows addressed in the text in relation to CT → MC conversion (box 1) and to ISC within the CT manifold (box 2). Oscillations contributing to the fast TA dynamics are also visible (see also Fig. 4a).

Wide-band oTA spectra were collected for (^CF3^L^1^)_2_Fe using a previously described^28^ in-house set-up. Optimized experimental conditions made use of polarization filtering to minimize excitation scatter and ultrathin (100 µm) cuvettes to avoid artifacts (see SI for full details). Toluene solutions of (^CF3^L^1^)_2_Fe (∼0.3 mM) were pumped using 600 nm excitation and probed covering a spectral range of 370 to 1200 nm (Fig. 2). In nearly all investigated spectral regions, we observed strong oscillations and rich spectral dynamics on both very fast and slow time scales. We first analyzed the oTA spectral dynamics to identify the sequence of excited-state transformations and evaluate their characteristic times. This qualitative analysis made use of spectroelectrochemistry to identify spectroscopic signatures of any charge-transfer excited states and the optical properties of (^CF3^L^2^)_2_Fe to simulate the quintet ligand-field state. Having thus produced a model of the excited-state dynamics of (^CF3^L^1^)_2_Fe, we inputted this information into global fitting to quantify all variables in the model. An important outcome of our fitting was that we were able to extract three oscillatory components that we correlate to excitation-induced coherent vibrational dynamics (*vide infra*).

### Qualitative analysis

**Fig. 3:**
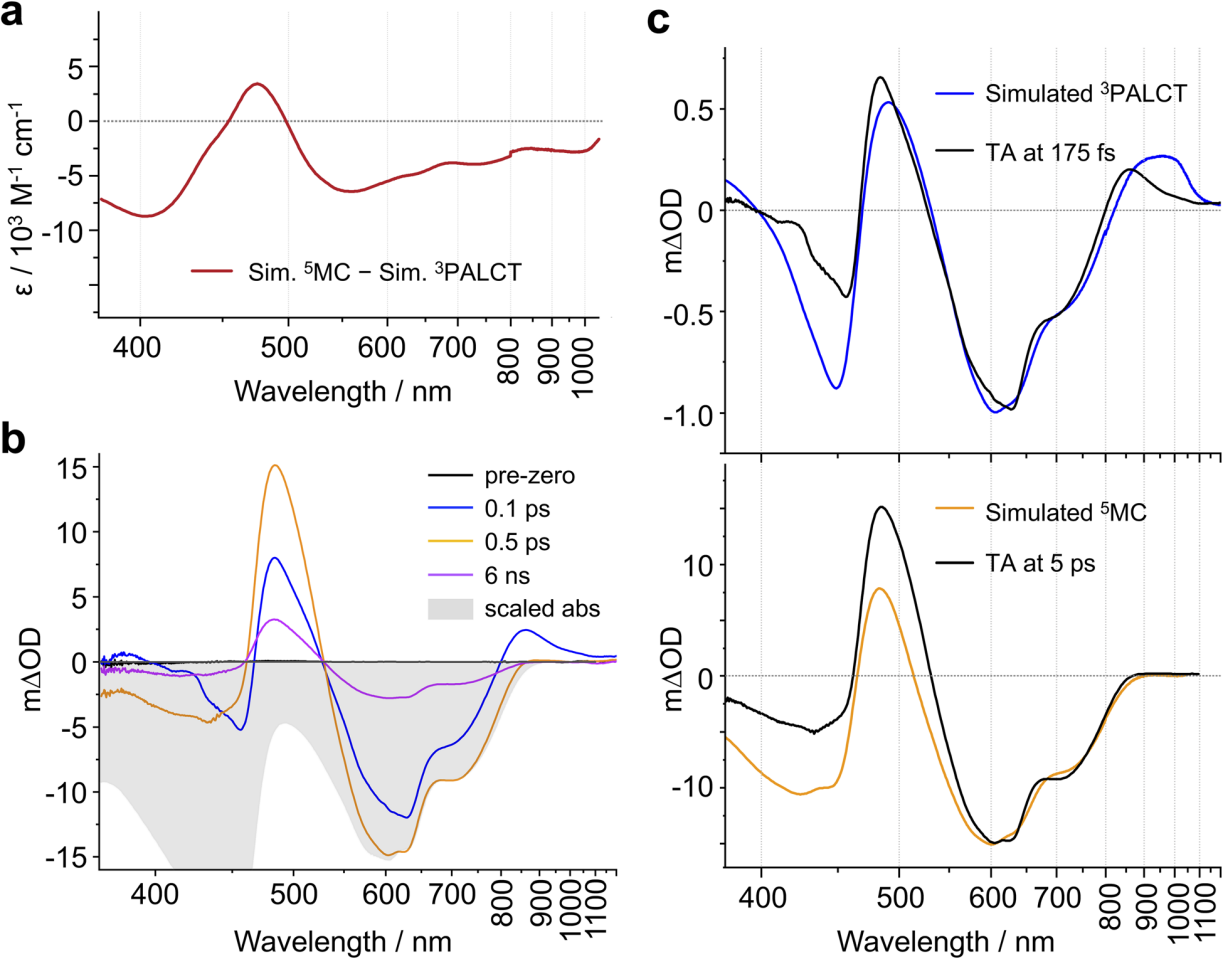
(a) The calculated difference between the differential spectrum simulated for a ^5^MC excited state using (^CF3^L^2^)_2_Fe and (^CF3^L^1^)_2_Fe and the simulated spectrum for a ^3^PALCT state simulated using spectroelectrochemistry. (b) TA spectra of (^CF3^L^1^)_2_Fe in deaerated toluene at selected delay times (*λ*_excitation_ = 600 nm). The steady-state absorption spectrum is scaled to the TA spectra at 0.5 ps. (c) TA spectra (*λ*_excitation_ = 600 nm) in deaerated toluene (black) at delay times of 175 fs (top) and 5 ps (bottom) are overlaid with scaled simulated difference spectra.

**Fig. 4:**
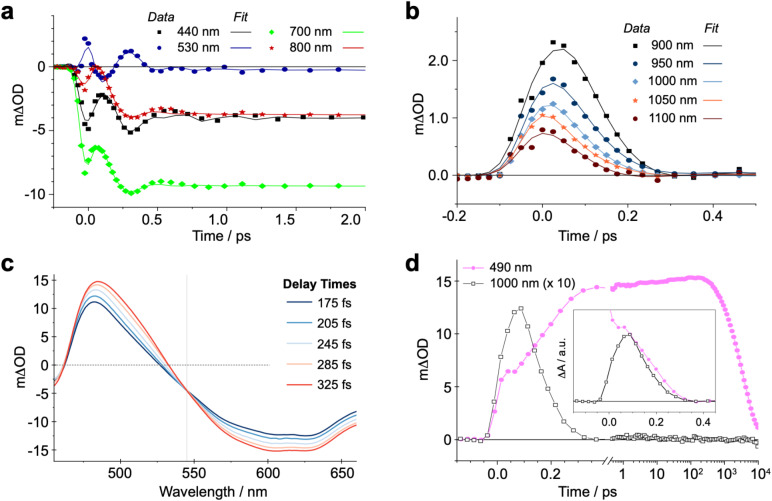
Transient absorption data of (^CF3^L^1^)_2_Fe in deaerated toluene at ultrafast timescales (*λ*_excitation_ = 600 nm): (a) measured data shown with sin(*ωt*) fit curves representing different parts of the covered spectral region, with the contribution of oscillations clearly visible at all kinetics. (b) Measured NIR data and lines from the sin(*ωt*) fit representing the dynamics of ^3^PALCT-to-^3^MC transition. Oscillations do not contribute to kinetics in this spectral region with large amplitude. (c) TA spectra at selected delay times connected to the ^3^MC → ^5^MC transition. The vertical line represents an isosbestic point. (d) Measured kinetics at 490 nm (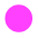) and 1000 nm (□). The signal at 1000 nm is amplified 10× for visual clarity, with the inset comparing the kinetics qualitatively through locking them in amplitude at two time points to distinguish their dynamics within the limits of the observed signal-to-noise.

In our qualitative analysis, we divide the observed spectral evolution into two time-domains: fast dynamics covering from the instrument response function (IRF = 100 fs) to <5 ps, and slow(er) dynamics at delay times beyond 5 ps when the influence of oscillations has vanished, up to ∼10 ns (Fig. 2 and 4a). The excited-state dynamics on the ns timescale of (^CF3^L^1^)_2_Fe are dominated by a synchronous decay to zero differential absorption signal in the entire spectral region, with a major time component of ∼3.5 ns (Fig. S11). Based on insights from spectroelectrochemistry and our ^5^MC model complex (^CF3^L^2^)_2_Fe, we can demarcate these temporal regimes as representing an initially formed, short-lived PALCT excited state and a long-lived ^5^MC state (Fig. 3). On the faster timescale, a relatively broad, strong absorption band at ∼800–1200 nm is present at early delay times (Fig. 3b), mirroring simulations of the CT excited state owing to the characteristic absorption features of oxidized [(^CF3^L^1^)_2_Fe]^+^ (Fig. 3c).^24^ In the oTA data at longer delays (0.5 ps and beyond ∼ ns), and in the ^5^MC spectrum simulated by taking the difference of the absorption spectra of the high-spin quintet (^CF3^L^2^)_2_Fe and the low-spin singlet (^CF3^L^1^)_2_Fe, this feature is absent. In addition, an initially positive oTA signal observed at higher energy (<400 nm) fully converts into a negative absorption feature (bleach) only after ∼300 fs. A similar bleach appears in the simulated ^5^MC spectrum, contrasting with the positive feature below 400 nm in the simulated CT spectrum (Fig. 3c). With contributions from oscillations between 900 and 1150 nm nearly absent at this stage, we resolve a decay component of ∼100 fs for the NIR feature (Fig. 2 and 4b), which we assign to the PALCT → MC transition.

Prior to the ∼100 fs decay component, we can discern an even faster process. While these dynamics are limited by the IRF and therefore difficult to quantify unambiguously, we can discriminate negative features between 420-500 and 600–900 nm, and a positive feature at 520–560 nm (Fig. 2, 4a, S11 and S16) that decay significantly faster than the 100 fs time constant we assign to the PALCT → MC transition with its distinctive NIR kinetics (Box 1 in Fig. 2; see also Fig. 4b and d). We rule out solvent contributions/cuvette artefacts as the origin of the ultrafast features thanks to the combination of (^CF3^L^1^)_2_Fe's strong optical transitions, the reduced thickness of the cuvette windows, and the shorter solution path length (both 100 µm). Collectively, these optimized measurement conditions yield a much stronger solute signal compared to the solvent response (Fig. S7). As this resolved spectral evolution precedes the PALCT-to-MC transition (*vide supra*), we assign these ultrafast dynamics to intersystem crossing (ISC) *within* the PALCT manifold – *i.e.*, from a ^1^PALCT state to the ^3^PALCT state. Considering the underlying electronic transition responsible for these features, while we excite the molecule at a higher energy compared to its lowest possible energy absorption (*λ*_excitation_ = 600 nm *cf. λ*_absorption_ to ∼900 nm; Fig. 1d), intramolecular vibrational energy redistribution (IVR) is expected to occur on slower timescales.^33–35^ Moreover, IVR is typically accompanied by a spectral shift of both the ESA and stimulated emission (if any) as the molecule relaxes,^36^ which we do not observe *prior* to the 100 fs delay in our oTA spectral evolution (Fig. S6b and c). As discussed further in the SI, we do not believe these dynamics are significantly influenced by the observed oscillations.

Following ^1^PALCT → ^3^PALCT ISC (∼50 fs) and the subsequent ^3^PALCT → MC transition (∼100 fs), another fast process can be observed between 150–400 fs. This next step in the spectral evolution of the photoexcited (^CF3^L^1^)_2_Fe is identified by a clear isosbestic point at 545 nm which persists across delay times between 175–325 fs (Fig. 4c). The changes in the oTA spectra before and after the isosbestic point are obvious, if not dramatic. Comparing the kinetics of the spectral changes associated with the isosbestic point—focusing at 490 nm, where the maximum amplitude rise is observed in the isosbestic point region—with the dynamics of the positive feature observed between ∼800–1100 nm (which represents the ^3^PALCT → MC transition; Fig. 4d) makes clear that, despite overlapping in time to a certain extent with the ∼100 fs decay component, the isosbestic point appears largely after depopulation of the ^3^PALCT state but before the long-lived ^5^MC excited state is fully established, with a time component of ∼250 fs. The inset of the Fig. 4d, using only measured, unfitted data and locking these data in amplitude at two time points, clearly distinguishes these different dynamics within the limits of the observed signal-to-noise. We therefore associate this third process with the ^3^MC → ^5^MC transition, assuming a quantitative ^1^MLCT → ^3^MLCT → ^3^MC → ^5^MC → ^1^GS trajectory with no relaxation to the ground state prior to population of the ^5^MC.

Having staked out the time periods for each of the four light-generated excited states of (^CF3^L^1^)_2_Fe (*i.e.*, ^1^PALCT → ^3^PALCT → ^3^MC → ^5^MC), we next turned our attention to the nature of the individual spectral profiles of each excited state. The main contributions to the oTA spectra are the loss of absorptive features associated with (^CF3^L^1^)_2_Fe's electronic ground state (*i.e.*, the ground-state bleach or GSB) and any excited-state absorptions (ESAs). As the shape of the GSB is known from the steady-state absorption spectrum, differences in the profiles observed by oTA mainly arise from differences in the shape of any ESAs. The shapes of the ESAs of individual electronic excited states can thus be semi-quantitatively extracted from the oTA data by subtracting the scaled ground-state absorption (GSA) spectrum from the oTA difference spectra at specific delay times. In subtracting the GSB, we aim at the absence of any remaining negative amplitude in the difference (due to under-scaling of the inverted absorption) and at the absence of any positive spectral features with the shape of the ground-state absorption (due to over-scaling). Scaling the GSA imposes a quantification factor for the excited-state concentration and the ESA spectra after subtraction are obtained in the absolute value of extinction. This subtraction is most reliable if, as for (^CF3^L^1^)_2_Fe, spectral features characteristic of the GSA are also evident in the GSB (to allow for suitable scaling) and if the shapes of the ESA spectra are substantially different from the GSA spectrum. To this latter point, the absorption spectrum of (^CF3^L^2^)_2_Fe (Fig. 1d) suggests that the ESA of the ^5^MC state (observed at delays >1 ps) should be quite different from the GSA and nearly negligible at wavelengths longer than 750 nm. Another assumption is that the excited state population evolves between consecutive excited states *en route* to ground-state recovery (GSR) rather than leaking back to the ground state. While multi-faceted deactivation dynamics are known to occur in both Fe(ii) and Fe(iii) chromophores,^23,37,38^ we do not observe any significant signatures of GSR faster than on the ns timescale (see SI for detailed discussion). Thus, the excited-state concentration is equal to the ‘concentration’ of absorbed photons, and the same GSA scaling factor can be used for subtraction from any oTA spectrum at delays after the end of the IRF and before substantial ground-state recovery has occurred. For (^CF3^L^1^)_2_Fe, we chose to scale the GSA to the negative oTA signal between 600–850 nm measured in the relaxed ^5^MC state (*i.e.*, the lowest-lying excited state) at a delay time of 2 ps. At this delay time, the ^5^MC state should be fully populated with all faster processes and oscillations complete, but GSR not yet commenced. Judging by the amplitude of GSB, we evaluate the fraction of excited molecules as 5 ± 0.25% and their concentration as 1 ± 0.05 ×10^−4^ M. This result is in perfect agreement with the concentration of absorbed excitation photons (1 ± 0.15 10^−4^ M) determined using the excitation fluency, the cuvette pathlength, and the sample optical density (see SI). For delay times shorter than the IRF, we account for incomplete excitation by reducing the GSB amplitude, calculating the reduction factor as the ratio of the IRF integrated up to a specified delay time relative to the integral of the entire IRF.

**Fig. 5:**
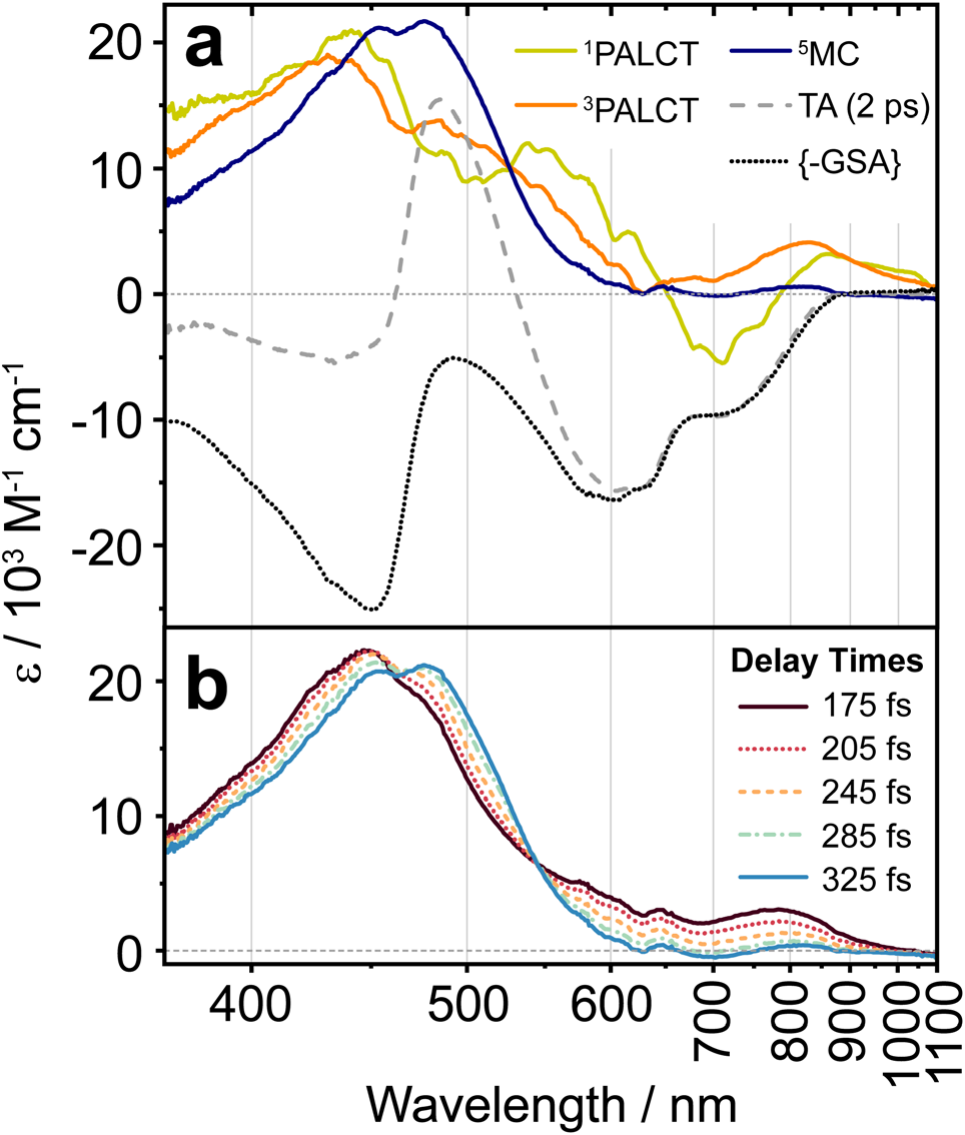
(a) ESA spectra of the individual identified excited states extracted *via* GSA subtraction and overlaid with the oTA spectrum at a delay of 2 ps and the scaled, inverted GSA spectrum of (^CF3^L^1^)_2_Fe in deaerated toluene. (b) ESA spectra identifying the isosbestic point at 540 nm associated with the ^3^MC-^5^MC transition, with delay times noted in the insert. The excitation wavelength in all cases was 600 nm.

For each of the ^1^PALCT, ^3^PALCT and ^5^MC excited states of (^CF3^L^1^)_2_Fe, we chose a representative delay time that presents the highest contribution of each state (Fig. 5a). This is easily done for the ^5^MC state but is more challenging for the ^1/3^PALCT states. For the initially formed ^1^PALCT excited state, we selected the oTA spectrum where the delay time between the pump and probe pulses is 0 ps, as this component decays much faster than the IRF and will have the largest amplitude close to the 0 ps delay. For the ^3^PALCT, we chose a delay of 75 fs as this time point corresponds to the maximum amplitude in the NIR kinetics (Fig. 4b). In the so-constructed ^1^PALCT spectrum there is some contribution of the ^3^PALCT spectrum, as well as minor contributions from the ^3/5^MC spectra. Correspondingly, the constructed ^3^PALCT spectrum contains minor contributions from the ^1^PALCT spectrum and some ^3/5^MC spectral contamination*.*Fig. 5b contains ESA constructions covering the time-period across which the isosbestic point assigned to the ^3^MC → ^5^MC is observed (175–325 fs). The ESAs of the ^3^MC and ^5^MC states are alike but sufficiently distinct to be differentiable. The constructed ESA of the ^5^MC state is clean as contributions of all other states can be neglected at 2 ps delay, whereas the ESA of the ^3^MC likely contains contributions of both the ^3^PALCT and ^5^MC states. This approach cannot, of course, fully discriminate spectra of different species in our decay model; appropriate global fitting is required to extract ‘species-associated spectra’ (SAS).

The ESA spectra extracted for the ^1^PALCT and ^3^PALCT excited states both show absorption between 800 and 1100 nm (Fig. 5a). The NIR absorption observed for the ^3^PALCT state agrees with the spectroelectrochemically generated reference spectra (Fig. 3). Interestingly, the ESA spectrum of the ^1^PALCT state displays a negative spectral feature at 700 nm. As the GSA has already been subtracted from the oTA data, the GSB should not contribute here and the negative signal can be reasonably assigned to stimulated emission (SE) from the ^1^PALCT state.^19^ Notably, this SE overlaps with the lowest energy absorption band. In our oTA experiments, we used an excitation wavelength of 600 nm. Based on prior TD-DFT studies,^24^ the transitions that most prominently contribute to (^CF3^L^1^)_2_Fe's absorption at this wavelength are of mixed PALCT and MLCT character, involving the filled HOMO/HOMO-1 {with Fe(3d)-N_amido_(2p) π* antibonding character} and the Fe-based HOMO-2, and vacant orbitals with localized C=N π* character (LUMO) and larger contributions from the C_6_ rings of the phenanthridine ligands (LUMO+4, LUMO+5; Fig. S27); the pure PALCT band is lower in energy. Excitation into this higher energy interconfigurational PALCT/MLCT band should still result in the population of the lowest energy ^1^PALCT excited state in accordance with Kasha-type behavior,^39^ from which radiative decay back to the *S* = 0 ground state would be spin-allowed. At the same time, an allowed absorption transition to an excited state implies emission from that same state is feasible. The competing quenching process is ISC to the ^3^PALCT, which is very fast (∼50 fs). If ISC is faster than downhill internal conversion between ^1^PALCT-type states (*i.e.*, within the singlet excited-state manifold), SE from the initially photo-populated ^1^PALCT state will predominate.

The intense ESA in the visible region observed for the ^5^MC state (Fig. 5a) is unusual for metal-centred excited states^40^ and warrants some discussion. High-spin (^CF3^L^2^)_2_Fe absorbs rather strongly at ∼480 nm (Fig. 1d; see Fig. S28 for comparison with ^5^MC ESA) and TD-DFT simulations^27^ suggest that this band arises from intraligand charge-transfer (ILCT) transitions, with electron density relocating from the N_amido_, phenanthridine-based C_6_ rings (Ar_phen_), and quinoline-based C_6_ rings (Ar_quinoline_) to vacant orbitals with localized C=N π* and Ar_phen_ π* character. Within the low energy tail at wavelengths beyond 500 nm, we find some weak MLCT transitions (*f*_osc_ < 0.01). We thus assign the strong ESA as ILCT in character, with only minor MLCT character at the low energy tail. With negligible metal contribution and comparably large ligand contributions, the energies of these transitions are not expected to differ significantly between the ^3^MC and ^5^MC excited states. This is supported by the ESA shapes extracted by the GSA subtraction treatment (Fig. 5b), showing only a small bathochromic shift to the ESA maximum across the isosbestic point spectral region. TD-DFT simulations of the absorption spectra using the optimized geometries of the ^3^MC and ^5^MC excited states of a closely related analogue (^Cl^L^1^)_2_Fe (with Cl groups in place of the CF_3_ substituents) reproduce this red-shift in the absorption on moving from *S* = 1 to *S* = 2 (Fig. S29). The minor differences in the ESA for what we assign as the ^3^MC excited state and that of the long-lived ^5^MC excited state may stem from the slight differences that are expected between the geometries of these two states, as reflected in our computations (Fig. S30). It is interesting to note the presence of a second (weak) ESA for the ^3^MC excited state appearing at ∼700–1000 nm. While we cannot rule out the ^3^PALCT state as its origin, a similar feature does appear in the TD-DFT simulated absorption spectrum of the ^3^MC excited state of (^Cl^L^1^)_2_Fe, as a low-energy tail that is absent in the simulated absorption spectrum of the ^5^MC excited state (Fig. S29). Considering our assignment of the ^3^MC → ^5^MC transition to the spectral evolution around the isosbestic point from 150–360 fs, we acknowledge this could instead arise from vibrational relaxation processes within the MC manifold, as we do observe a red shift and narrowing of the ESA over time. We note, however, that spectral shifts/narrowing arising from vibrational relaxation processes alone do not typically result in isosbestic points.^41^ Here, the oTA spectra may incidentally cross at the same wavelength point over several delay times during combined vibrational relaxation and simultaneous signal oscillations, but this scenario seems less probable than observation of a state-to-state transition (*e.g.*, ^3^MC → ^5^MC).

### Global fitting

In the above analysis, we were able to identify sequential conversion of the excited state from ^1^PALCT → ^3^PALCT → ^3^MC → ^5^MC. We next used this assignment, and the characteristic times of the state-to-state conversions obtained, as input for global fitting. To reduce the number of variables introduced, we followed a step-by-step procedure, applying the simplex algorithm. First, we successfully fit the chirp-corrected data on the slower timescale when the observed oscillations are over (see Fig. 2 and 4a). We then extended the fitted exponential decays to zero time, convoluted with the IRF, and subtracted this part from the measured chirp-corrected data. Residuals which exhibit clear oscillating features were then fit with an oscillatory model outside the pump-probe pulse overlap. These were again extended to zero, convoluted, and further subtracted from the data. The new residuals were fit to extract the next exponential decay, and the procedure repeated until the fit residuals appeared close to randomly distributed about the zero line. We then returned to the original chirp-corrected data and subtracted only the fitted oscillations to construct a new oscillation-free set of data. This data set was fit using the simplex Nelder–Mead algorithm to find the global minimum in the optimization. Finally, the model was optimized using the least-mean-square algorithm with all exponential decays and oscillations included, targeting the original data with the results of the previous steps used as initial guesses. The results of this final optimization are given in Table 1 and the SI. The upper and lower margins of every variable obtained *via* a *T*-test indicate that all components are well-defined with close to 5% accuracy.

**Table 1:** Results of the final optimized global fit of the oTA data including upper and lower margins for every variable (*T*-test)

Variable	Fit (lower/upper margins)
Oscillation 1 period/decay	546 fs (519 fs/574 fs)/134 fs (127 fs/140 fs)
Oscillation 2 period/decay	300 fs (285 fs/315 fs)/513 fs (488 fs/540 fs)
Oscillation 3 period/decay	402 fs (382 fs/422 fs) / 345 fs (328 fs/363 fs)
K1	30 fs (28.5 fs/31.5 fs)
K2	100 fs (95 fs/105 fs)
K3	250 fs (238 fs/263 fs)
K4	10.9 ps (10.4 ps/11.5 ps)
K5	138.6 ps (132 ps/145.9 ps)
K6	3.5 ns (3.3 ns/3.7 ns)

In addition to the overall depopulation of the excited state on the ∼3.5 ns timescale and the very fast processes occurring during the first 0.5 ps, global fitting provides two components with the characteristic times of ∼11 and ∼140 ps. The origin of these components is not abundantly clear: they are characterized by minor changes in the oTA spectra and SAS comparable to the spectrum of ^5^MC state. While the shorter component could be associated with vibrational relaxation, the slower one appears to be too slow for VR. As the same dynamics were observed at both magic-angle and perpendicular relative polarizations of the probe and pump beams, the ∼140 ps component cannot be associated with solvent reorientation dynamics. We tentatively associate this process with molecular conformational relaxation. Similar dynamics have been recently described.^42^ The successful global fit of the recorded data provides SAS of the intermediate states after subtraction of the GSA (Fig. S12, S17 and S22). These spectra are in good agreement with spectra obtained at later delay times by subtraction of the GSB from the oTA spectra representing the maximum population of each identified intermediate state (Fig. 5a). The SAS representing early times would reasonably be expected to differ, as the early oTA spectra are likely contaminated by the following and preceding states in the excited-state conversion chain. In particular, the SAS of the ^1^PALCT state is substantially different, as the decay time of this state is significantly shorter than the IRF. Furthermore, this shortest component is bound to be the most sensitive to the uncertainty of the chirp definition as well as to solvent and cuvette artefacts. Overall, the ground-state recovery time (3.5 ± 0.2 ns) is in agreement with prior TA^24,25^ and XES^27^ measurements (Tables 2 and S1–S9). The very fast decay time of the ^1^PALCT state (30 ± 1.5 fs; Table 2) is difficult to determine accurately, although the fit appears quite exact as the error bars determined *via T*-test are small (Table 1). This decay time is in agreement with previously reported ^1^MLCT → ^3^MLCT intersystem crossing (ISC) times of <50 fs in iron(ii) complexes.^19,20^ The ^3^PALCT decay time of 100 ± 5 fs is similarly in good agreement with time-resolved X-ray emission spectroscopy measurements^27^ where a time component of ∼120 fs was determined for the decay of the ^3^PALCT state of (^CF3^L^1^)_2_Fe.

**Table 2:** Decay components identified in various studies of (^CF3^L^1^)_2_Fe in deaerated toluene at room temperature. All parameters and all spectra were fitted

Technique	Narrow-band oTA (490–800 nm)	trXES (two-state kinetic model)	trXES (three-state kinetic model)	Wide-band oTA (370–1200 nm)
^1^PALCT	not resolved	not resolved	not resolved	∼30^a^ fs
^3^PALCT	2.7^b^ ns	122 fs	113 fs	∼100 fs
^3^MC	—b	—	21 fs	∼250 fs
^5^MC	—b	3.3 ns	3.3 ns	3.5 ns
Reference	24	27	27	this work

a Faster than pulse duration.

b The long-lived excited state was initially assigned as PALCT, later revised to a ^5^MC excited state.

For [Fe(bpy)_3_]^2+^, time-resolved iron Kβ fluorescence spectroscopy was used to delineate a sequential ^1,3^MLCT → ^3^MC → ^5^MC relaxation trajectory with a 150 ± 50 fs time constant for the ^1,3^MLCT decay to the ^3^MC state, and a 70 ± 30 fs time constant for the subsequent population of a long-lived ^5^MC state.^21^ The rate of ^3^MC decay was found to exceed the rate of ^3^MC formation, explaining why similar timeframes were observed for the decay of signals ascribed to the ^1,3^MLCT states and the rise of the ^5^MC state. For (^CF3^L^1^)_2_Fe, the oTA spectrum at 250 fs is dominated by the oTA signal from the ^3^MC state. This reflects substantial buildup of this intermediate following ^3^PALCT decay, resulting in a clear isosbestic point for the ^3^MC → ^5^MC transition. Our recent time-resolved X-ray emission spectroscopy (trXES) study on (^CF3^L^1^)_2_Fe considered the possibility that a transient ^3^MC state with a 21 fs lifetime is populated before the ^5^MC state *via* a three-step, consecutive-decay model.^27^ This three-step spectrotemporal model, however, did not improve the data fitting to a statistically meaningful extent. In this study, by means of oTA spectroscopy we clearly identify the ^3^MC → ^5^MC transition on the ultrafast time scales through the observation of an isosbestic point at 545 nm and quantify the characteristic time of this transition as 250 fs.

### Oscillations

As noted, distinct oscillations are evident in the oTA data (Fig. 2 and 4a), persisting up to 1.5 ps. Prior to any fitting, we emphasize that we detect prominent oscillations in the spectral region between 600 and 900 nm where the GSB is the dominant contribution to oTA spectra collected after a ∼0.3 ps delay. Thus, we conclude that these oscillations at least partially occur in the ground state. Further analysis required global fitting. We note that several fitting models for oscillations worked reasonably well, highlighting the argument that fitting without a model which is certified by qualitative analysis of measured data, calculations, or previous publications, for a complex set of processes in a studied system, can lead to inconclusive or even misleading results. We therefore based our quantitative analysis of oscillations on several well-accepted models previously reported in the literature. In each model, three oscillations were needed to fit the data.

**Fig. 6:**
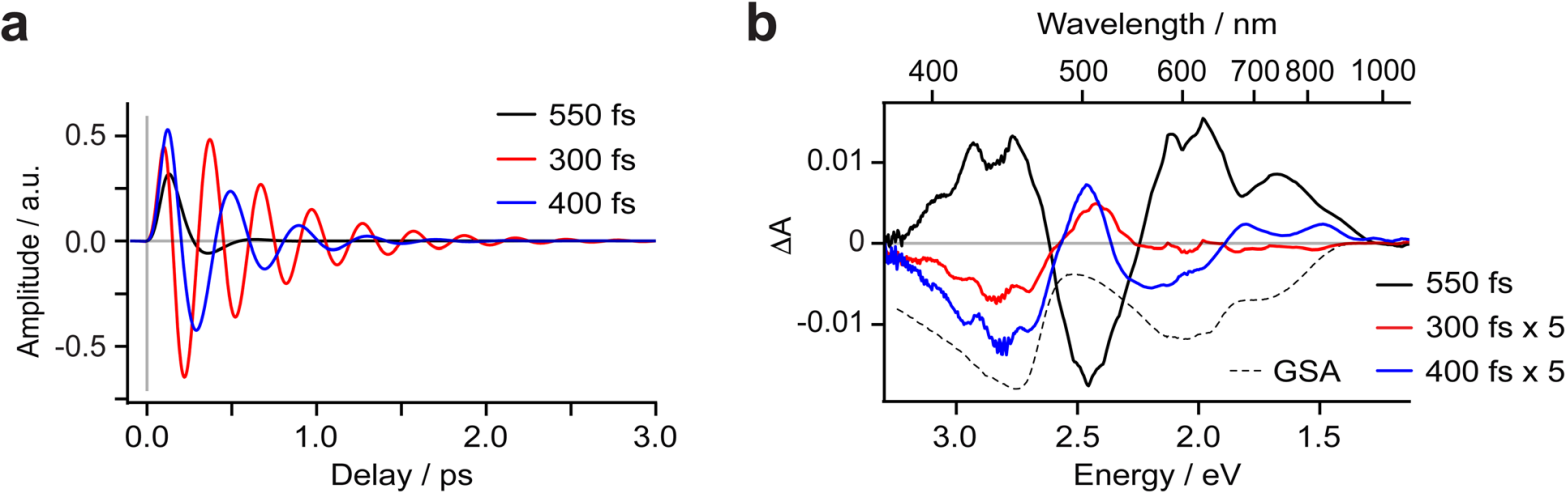
Oscillations and amplitude spectra fitted using sin(*ωt*) functions with the oscillation periods given in the legends (see Fig. S23 for cos(*ωt*) and delayed cos(*ωt*) fits). The inverted, scaled ground-state absorption (GSA) is provided for comparison.

Observation of oscillations in the ‘GS-only’ part of the oTA spectra implies they originate from an impulsive Raman process. Such ground-state oscillations would be characterized by a sin(*ωt*) functional form as excitation-induced oscillations instigate with a vibrational wave packet close to the equilibrium ground-state molecular geometry; that is, with minimal molecular distortion.^43–45^ Fitting with a sin(*ωt*) functional form works well and returns three oscillation modes (see Fig. 6a, b, Table S3 and Fig. S10, S11 and S23). Two of these exhibit relatively large amplitude between 600–1000 nm, while all three show large amplitude in the rest of the studied spectral range with the exception of the very red (>950 nm) and the very blue (<395 nm). The fit returns substantially different periods for the three oscillations (550, 300 and 400 fs corresponding to ∼60, 110 and 80 cm^−1^; Fig. S23 and Table 1). The dumping times are comparable to the oscillation periods for two oscillations (345 and 515 fs) and much shorter (135 fs) for the most intense. Further analysis of the fit results suggests that the amplitude spectrum of the oscillation resembles the ground-state absorption spectrum (see Fig. S24 and S25). This implies that the vibrations coupled to these oscillations drive the molecule in/out of the Frank–Condon active geometry.

We also applied a cos(*ωt*) functional form to fit the oscillations and note that this fitting works equally well (see Fig. S23; Table S6, Fig. S15 and S16c). Such a functional form would suggest oscillations that are initiated by molecular distortion with a maximum amplitude reached on generation, in this case, in the excited state under optical excitation.^28,38^ The relatively short decays of these oscillations might mean these are dampened by the CT-to-MC excited state conversion. While mathematically feasible, this assignment conflicts with the observation of oscillations in the ‘GSB-only’ region of the oTA spectra and the substantial amplitude spectra of the fitted oscillations in this same spectral region. We also attempted to fit oscillations following the conventional model of oscillations generated in the MC manifold by fast CT → MC conversion.^46–50^ Practically, such oscillations should be fit by a cos(*ωt*) function to represent the molecular distortion, with a maximum amplitude delayed by the CT → MC conversion. We applied this fit using a sum of cos(*ωt*) functions delayed compared to the IRF. The best fit, although marginally worse than the two previous fits, requires an additional rise time of 160 fs (Table S9). This delay in the oscillation build-up correlates fairly well with the decay of the ^3^PALCT population and thus could support the assignment of ‘oscillations generated by the CT → MC transition’. The amplitude spectra of the fitted oscillations, however, clearly demonstrate the oscillations have a prominent contribution in the ‘GSB-only region’ of the oTA data (Fig. S23). This is a strong argument to discard this model of oscillations despite its support from literature and apparent correlation with the previously settled population dynamics. We therefore conclude the oscillations originate in the ground state.

## Conclusions

Optical TA spectroscopy is arguably unmatched in combining accessibility and detail regarding excited-state decay cascades of transition metal coordination complex photosensitizers.^40^ Merging variable relative polarization of excitation and probe beams, and supported by *ab initio* calculations, this technique allows detailed characterization of photoinduced processes in a wide range of materials.^51^ In this work, we exploit high temporal resolution with very high signal-to-noise and cover a broad spectral range (370 to 1200 nm) to revisit the excited state dynamics of iron(ii)-amido chromophores. Expanding the spectral range allowed us to definitively observe formation of a charge-transfer (here, PALCT) excited state through appearance of a low-energy excited-state absorption (ESA) in the NIR, and its decay with a ∼100 fs decay component into a nanosecond-lived MC excited state, in agreement with recent insights from time-resolved X-ray emission spectroscopy experiments.^27^ In the measured data, we identify coherent oscillations and assign them to impulsive Raman-induced vibrations in the ground state. By global fitting, we extract three vibrations at 60, 80 and 110 cm^−1^.

The ultrafast time resolution of the measurements also made it possible to resolve the previously elusive transient ^1^PALCT state which undergoes ISC to the ^3^PALCT, from which spin-conserved, stimulated emission (SE) can be seen. Within the MC state manifold, we furthermore observe an isosbestic point in the spectral evolution that we interpret in terms of the ^3^MC → ^5^MC transition with a decay component of ∼250 fs. With the help of reference spectra obtained using spectroelectrochemistry and tailored synthetic modifications to the studied chromophores, we can resolve transitions between states of different spin multiplicity in both the CT and MC manifolds and fully identify the sequential four-state decay cascade (^1^PALCT → ^3^PALCT → ^3^MC → ^5^MC) that precedes ground-state recovery for (^CF3^L^1^)_2_Fe, mirroring the ^1^MLCT → ^3^MLCT → ^3^MC → ^5^MC pathway understood to dominate the decay cascades of d^6^ polypyridyl chromophores more generally.^21^

## Author contributions

The manuscript was written through contributions of all authors. All authors have given approval to the final version of the manuscript. Christina Wegeberg: conceptualization, data curation, formal analysis, investigation, visualization, writing – original draft, writing – review & editing. Baldeep K. Sidhu: conceptualization, data curation, formal analysis, investigation, visualization, writing – original draft, writing – review & editing. Pavel Chábera: formal analysis, investigation, visualization. Jens Uhlig: formal analysis, investigation, methodology, visualization. Rory A. Cowin: investigation, visualization. Julia A. Weinstein: investigation, visualization. Petter Persson: conceptualization, data curation, formal analysis, funding acquisition, supervision, resources, writing – review & editing. Arkady Yartsev: conceptualization, data curation, formal analysis, funding acquisition, supervision, resources, investigation, methodology, visualization, writing – review & editing. David E. Herbert: conceptualization, data curation, formal analysis, funding acquisition, supervision, resources, visualization, writing – review & editing, project administration.

## Conflicts of interest

There are no conflicts to declare.

## Supplementary Material

SC-017-D5SC06464C-s001

## Data Availability

The data underlying this study are available in the published article and the provided supplementary information files (SI). The latter are provided, free of charge: experimental details including materials and methods and a full description of the oscillation removal protocol, additional supporting figures, and discussion (PDF); the full data package (.zip). See DOI: https://doi.org/10.1039/d5sc06464c.
