# Supplementary material for: Resolving consecutive excited-state evolution in Fe-amido chromophores by wide-band optical transient absorption spectroscopy
Source: Chem Sci. 2026 Jan 15;17(12):6125–37. doi: 10.1039/d5sc06464c (PMC12865702; doi:10.1039/d5sc06464c)
Supplement: SC-017-D5SC06464C-s001 [file SC-017-D5SC06464C-s001.pdf]

Supplementary Information (SI) for Chemical Science.
This journal is © The Royal Society of Chemistry 2026

Supporting Information

for

Resolving Consecutive Excited State Evolution in Photoexcited Fe-Amido Chromophores by Wide-Band Optical Transient Absorption Spectroscopy

Christina Wegeberg,[a,†,‡] Baldeep K. Sidhu,[b,‡] Pavel Chábera,[a,†] Jens Uhlig,[a] Rory A. Cowin,[c] Julia A. Weinstein,[c] Petter Persson,[d] Arkady Yartsev[a]*, David E. Herbert[b]**

[a] Division of Chemical Physics, Department of Chemistry, Lund University, 22100 Lund, Sweden; *arkady.yartsev@chemphys.lu.se

[b] Department of Chemistry and the Manitoba Institute for Materials, University of Manitoba, 144 Dysart Road, Winnipeg, Manitoba R3T 2N2, Canada; *david.herbert@umanitoba.ca

[c] Department of Chemistry, University of Sheffield, Sheffield S3 7H, United Kingdom

[d] Division of Computational Chemistry, Department of Chemistry, Lund University, 22100 Lund, Sweden; *petter.persson@compchem.lu.se

[†] Current affiliations: Department of Chemistry, Physics and Pharmacy, University of Southern Denmark, 5230 Odense M, Denmark (CW); Department of Physics, College of Science, United Arab Emirates University, P.O. Box 15551, Al Ain, United Arab Emirates (PC)

[‡]These authors contributed equally to this work

TABLE OF CONTENTS

MATERIALS **5**

INSTRUMENTATION AND METHODS **5**

OPTICAL TRANSIENT ABSORPTION **6**

SPECTROELECTROCHEMISTRY **8**

FIGURE S1. SPECTROELECTROCHEMICALLY COLLECTED UV-VIS-NIR ABSORPTION SPECTRA OF THE OXIDATION OF $(^{CF3}\mathbf{L^1})_2$FE, WITH OXIDATIVE POTENTIALS APPLIED FROM -0.20 V TO 0.145 V VS. AG WIRE. 8

FIGURE S2. SPECTROELECTROCHEMICALLY COLLECTED UV-VIS-NIR ABSORPTION SPECTRA OF THE REDUCTION OF $(^{CF3}\mathbf{L^1})_2$FE, WITH REDUCTIVE POTENTIALS APPLIED FROM -0.70 V TO -1.05 V VS. AG WIRE. 8

FIGURE S3. CALCULATED DIFFERENCE SPECTRUM FOR THE PALCT EXCITED STATE OF $(^{CF3}\mathbf{L^1})_2$FE (BLACK SOLID TRACE), OVERLAID WITH THE INDIVIDUAL DIFFERENCE SPECTRA FOR THE OXIDIZED COMPLEX (RED DOTTED TRACE) AND THE REDUCED COMPLEX (BLUE DOTTED TRACE). 9

FIGURE S4. SIMULATED DIFFERENCE SPECTRA FOR THE PALCT EXCITED STATE (TOP) AND ^{5}MC EXCITED STATE (BOTTOM) FOR $(^{CF3}\mathbf{L^1})_2$FE. 10

OTA SUPPORTING DISCUSSION AND FIGURES **11**

FIGURE S5. (A) NIR OTA SPECTRA AND (B) KINETIC TRACES FOR $(^{CF3}\mathbf{L^1})_2$FE ($\lambda_{EXCITATION}$ = 780 NM). 11

FIGURE S6. TA SPECTRA OF $(^{CF3}\mathbf{L^1})_2$FE IN DEAERATED TOLUENE AT THE ULTRAFAST TIME SCALES, SEE DELAY TIMES IN INSERTS. 13

FIGURE S7. MEASURED KINETICS AT SELECTED WAVELENGTHS (SEE INSERT) FOR $(^{CF3}\mathbf{L^1})_2$FE IN DEAERATED TOLUENE (SYMBOLS) AND THE CORRESPONDING SOLVENT RESPONSE (DASHED LINES, LABELLED "S" IN LEGEND). 14

OSCILLATIONS AND GLOBAL FITTING **15**

CONTRIBUTION OF OSCILLATIONS TO FAST COMPONENTS **15**

STEPWISE GLOBAL FITTING **16**

TABLE S1. PARAMETERS OBTAINED FOR MODEL A (BEST R^2 = 0.9991 20

TABLE S2. PARAMETERS OBTAINED FOR MODEL B (BEST R^2 = 0.99948). FROZEN OSCILLATIONS PARAMETER AND ALL SPECTRA EXTRACTED. 20

TABLE S3. PARAMETERS OBTAINED FOR MODEL C (BEST R^2 = 0.99956). ALL PARAMETERS AND SPECTRA WERE FITTED. 21

FIGURE S8. MEASURED, MODELLED AND 10X THE DIFFERENCE SHOWN AS TWO-DIMENSIONAL MATRICES. ITERATIVE FITTING STEP AS DESCRIBED IN STEP 8 IN THE PROCEDURE ABOVE USING A SINUS FUNCTION FOR MODELLING THE OSCILLATIONS. NOTE: FOR THIS FIT, THE OSCILLATIONS WERE REMOVED FROM THE DATA AND MODEL PRIOR TO FITTING. 22

FIGURE S9. MEASURED, MODELLED, AND 10X THE DIFFERENCE SHOWN AS TWO-DIMENSIONAL MATRICES. FITTING WITH OSCILLATORY TEMPORAL PARAMETERS FIXED, BUT SPECTRA ALLOWED TO VARY AS DESCRIBED IN STEP 9 IN THE PROCEDURE ABOVE USING A SINUS FUNCTION FOR MODELLING THE OSCILLATIONS. 23

FIGURE S10. MEASURED, MODELLED, AND 10X THE DIFFERENCE SHOWN AS TWO-DIMENSIONAL MATRICES. FITTING WITH ALL PARAMETERS ALLOWED TO VARY AS DESCRIBED IN STEP 10 IN THE PROCEDURE ABOVE USING A SINUS FUNCTION FOR MODELLING THE OSCILLATIONS. 24

FIGURE S11. KINETIC DEVELOPMENT OF THE DATA FOR $(^{CF3}\mathbf{L^1})_2$FE. DOTS ARE MEASURED AND CHIRP CORRECTED DATA POINTS. THE LINES ORIGINATE FROM (A) STEP 8 IN THE DESCRIPTION ABOVE; (B) AND (C) STEP 9 AND 10 IN THE DESCRIPTION ABOVE. IN THIS MODEL, THE OSCILLATIONS WERE MODELED USING A SINUS FUNCTION. 25

FIGURE S12. EXTRACTED SPECIES-ASSOCIATED SPECTRA FROM THE FITS FOR $(^{CF3}\mathbf{L^1})_2$FE USING A SINUS FUNCTION. A): KINETIC MODELLING IN STEP 8 WITH REMOVED OSCILLATIONS AND LEAST-SQUARE OPTIMIZER. B) KINETIC AND SPECTRAL MODELLING IN STEP 9 WITH LOCKED OSCILLATION TEMPORAL PARAMETER. C) FREELY REFINING TEMPORAL AND OSCILLATOR PARAMETERS. FROM THE EXTRACTED SAS SPECTRA, THE GROUND STATE MEASURED

ABSORPTION SPECTRA WERE SUBTRACTED, AFTER IT WAS SCALED TO THE AVERAGE INTENSITY OF THE 10-15PS FEATURE BETWEEN 600 AND 700NM. 26

TABLE S4. PARAMETERS OBTAINED FOR MODEL A (BEST $R^2 = 0.9991$). SEPARATELY FITTED OSCILLATION WERE SUBTRACTED AND STANDARD KINETIC FITTED SPECTRA EXTRACTED. 27

TABLE S5. PARAMETERS OBTAINED FOR MODEL B (BEST $R^2 = 0.99949$). FROZEN OSCILLATION PARAMETER, ALL SPECTRA EXTRACTED. 27

TABLE S6. PARAMETERS OBTAINED FOR MODEL C (BEST $R^2 = 0.99956$). ALL PARAMETERS AND ALL SPECTRA FITTED. 28

FIGURE S13. MEASURED, MODELLED, AND 10X THE DIFFERENCE SHOWN AS TWO-DIMENSIONAL MATRICES. ITERATIVE FITTING STEP AS DESCRIBED IN STEP 8 IN THE PROCEDURE ABOVE USING A COSINE FUNCTION FOR MODELLING THE OSCILLATIONS. NOTE THAT FOR THIS FIT THE OSCILLATIONS WERE REMOVED FROM THE DATA AND MODEL PRIOR TO FITTING. 29

FIGURE S14. MEASURED, MODELLED, AND 10X THE DIFFERENCE SHOWN AS TWO-DIMENSIONAL MATRICES. FITTING WITH OSCILLATORY TEMPORAL PARAMETERS FIXED, BUT SPECTRA ALLOWED TO VARY AS DESCRIBED IN STEP 9 IN THE PROCEDURE ABOVE USING A COSINE FUNCTION FOR MODELLING THE OSCILLATIONS. 30

FIGURE S15. MEASURED, MODELLED, AND 10X THE DIFFERENCE SHOWN AS TWO-DIMENSIONAL MATRICES. FITTING WITH ALL PARAMETERS ALLOWED TO VARY AS DESCRIBED IN STEP 10 IN THE PROCEDURE ABOVE USING A COSINE FUNCTION FOR MODELLING THE OSCILLATIONS. 31

FIGURE S16. KINETIC DEVELOPMENT OF THE DATA FOR $(^{CF3}\mathbf{L^1})_2Fe$. DOTS ARE MEASURED AND CHIRP CORRECTED DATAPOINTS. THE LINES ORIGINATE FROM (A) STEP 8 IN THE DESCRIPTION ABOVE; (B) AND (C) STEP 9 AND 10 IN THE DESCRIPTION ABOVE. IN THIS MODEL, THE OSCILLATIONS WERE MODELED USING A COSINE FUNCTION. 32

FIGURE S17. EXTRACTED SPECIES-ASSOCIATED SPECTRA FROM THE FITS FOR $(^{CF3}\mathbf{L^1})_2Fe$ USING A COSINE FUNCTION. A): KINETIC MODELLING IN STEP 8 WITH REMOVED OSCILLATIONS AND LEAST-SQUARE OPTIMIZER. B) KINETIC AND SPECTRAL MODELLING IN STEP 9 WITH LOCKED OSCILLATION TEMPORAL PARAMETER. C) FREELY REFINING TEMPORAL AND OSCILLATOR PARAMETERS. FROM THE EXTRACTED SAS SPECTRA, THE GROUND STATE MEASURED ABSORPTION SPECTRA WERE SUBTRACTED, AFTER IT WAS SCALED TO THE AVERAGE INTENSITY OF THE 10-15 PS FEATURE BETWEEN 600 AND 700NM. 33

TABLE S7. PARAMETERS OBTAINED FOR MODEL A (BEST $R^2 = 0.9991$). SEPARATELY FITTED OSCILLATIONS WERE SUBTRACTED AND STANDARD KINETIC FITTED SPECTRA EXTRACTED. 34

TABLE S8. PARAMETERS OBTAINED FOR MODEL B (BEST $R^2 = 0.99936$). FROZEN OSCILLATION PARAMETER, ALL SPECTRA EXTRACTED. 34

TABLE S9. PARAMETERS OBTAINED FOR MODEL C (BEST $R^2 = 0.99937$). ALL PARAMETERS AND ALL SPECTRA FITTED. 35

FIGURE S18. MEASURED, MODELLED, AND 10X THE DIFFERENCE SHOWN AS TWO-DIMENSIONAL MATRICES. ITERATIVE FITTING STEP AS DESCRIBED IN STEP 8 IN THE PROCEDURE ABOVE USING A COSINE FUNCTION FOR MODELLING THE OSCILLATIONS. NOTE THAT FOR THIS FIT THE OSCILLATIONS WERE REMOVED FROM THE DATA AND MODEL PRIOR TO FITTING. 36

FIGURE S19. MEASURED, MODELLED, AND 10X THE DIFFERENCE SHOWN AS TWO-DIMENSIONAL MATRICES. FITTING WITH OSCILLATORY TEMPORAL PARAMETERS FIXED, BUT SPECTRA ALLOWED TO VARY AS DESCRIBED IN STEP 9 IN THE PROCEDURE ABOVE USING A COSINE FUNCTION FOR MODELLING THE OSCILLATIONS. 37

FIGURE S20. MEASURED, MODELLED, AND 10X THE DIFFERENCE SHOWN AS TWO-DIMENSIONAL MATRICES. FITTING WITH ALL PARAMETERS ALLOWED TO VARY AS DESCRIBED IN STEP 10 IN THE PROCEDURE ABOVE USING A COSINE FUNCTION FOR MODELLING THE OSCILLATIONS. 38

FIGURE S21. KINETIC DEVELOPMENT OF THE DATA FOR $(^{CF3}\mathbf{L^1})_2Fe$. DOTS ARE MEASURED AND CHIRP CORRECTED DATAPOINTS. THE LINES ORIGINATE FROM (A) STEP 8 IN THE DESCRIPTION ABOVE; (B) AND (C) STEP 9 AND 10 IN THE DESCRIPTION ABOVE. IN THIS MODEL, THE OSCILLATIONS WERE MODELED USING A COSINE FUNCTION. 39

FIGURE S22. EXTRACTED SPECIES-ASSOCIATED SPECTRA FROM THE FITS FOR $(^{CF3}\mathbf{L^1})_2Fe$ USING A COSINE FUNCTION. A): KINETIC MODELLING IN STEP 8 WITH REMOVED OSCILLATIONS AND LEAST-SQUARE OPTIMIZER. B) KINETIC AND SPECTRAL MODELLING IN STEP 9 WITH LOCKED OSCILLATION TEMPORAL PARAMETER. C) FREELY

REFINING TEMPORAL AND OSCILLATOR PARAMETERS. FROM THE EXTRACTED SAS SPECTRA, THE GROUND STATE MEASURED ABSORPTION SPECTRA WERE SUBTRACTED, AFTER IT WAS SCALED TO THE AVERAGE INTENSITY OF THE 10-15 PS FEATURE BETWEEN 600 AND 700NM. 40

FIGURE S23. TEMPORAL EVOLUTION OF THE OSCILLATIONS (LEFT COLUMN) AND SPECTRA ASSIGNED TO EACH OSCILLATION (RIGHT COLUMN). THE SELECTED MODELS ARE NAMED AS TITLE IN THE LEGEND. NOTE THAT SOME OF THE AMPLITUDES WERE AMPLIFIED, AS NOTED IN THE RESPECTIVE LEGENDS. 43

FIGURE S24. COMPARISON OF THE AMPLITUDE SPECTRUM OF THE OSCILLATIONS WITH 545 FS PERIOD AND SCALED GROUND-STATE ABSORPTION SPECTRUM. 44

FIGURE S25. COMPARISON OF THE AMPLITUDE SPECTRA OF OSCILLATIONS WITH PERIODS 400 AND 300 FS WITH SCALED GROUND-STATE ABSORPTION AND SMOOTHED AND SCALED DERIVATIVE OF THE ABSORPTION SPECTRUM.44

FIGURE S26. KINETIC DEVELOPMENT OF THE DATA FOR (^{CF3}**L**^{1})$_2$FE WITH THE FITTED OSCILLATIONS REMOVED FROM BOTH THE DATA AND THE FIT. THIS IS ESSENTIALLY THE SAME DATA AS IN FIGURE S11C) BUT NOW SHOWS ONLY THE KINETIC EVOLUTION OF THE DATA.......... 46

COMPUTATIONAL FIGURES AND SUPPORTING DISCUSSION 47

FIGURE S27. ELECTRON (GREEN) AND HOLE (BLUE) DENSITY DISTRIBUTION MAPS (ISOSURFACE = 0.002) FOR THE 2.05 EV (~600 NM) TRANSITION OF (^{CF3}**L**^{1})$_2$FE. 48

FIGURE S28. OVERLAY OF THE ESA ATTRIBUTED TO THE ^{5}MC STATE (325 FS) AND THE EXPERIMENTAL ABSORPTION SPECTRUM OF THE HIGH-SPIN MODEL COMPLEX (^{CF3}**L**^{2})$_2$FE. 48

FIGURE S29. COMPARISON OF (A) THE ESA SPECTRA ATTRIBUTED TO THE ^{3}MC STATE (175 FS) AND ^{5}MC STATE (325 FS) OF (^{CF3}**L**^{1})$_2$FE WITH (B) TD-DFT SIMULATIONS IN TOLUENE (SMD-D4-B3LYP-RIJCOSX-ZORA/ZORA-DEF2-TZVP+SARC/J; FWHM = 0.37 EV) OF THE ABSORPTION SPECTRA USING THE OPTIMIZED GEOMETRIES OF THE ^{3}MC AND ^{5}MC EXCITED STATES OF THE CLOSELY RELATED ANALOGUE (^{CL}**L**^{1})$_2$FE. 49

FIGURE S30. CALCULATED OCTAHEDRICITY PARAMETERS[27] FOR THE OPTIMIZED GEOMETRIES OF THE ^{3}MC AND ^{5}MC EXCITED STATES OF (^{CL}**L**^{1})$_2$FE, AND COMPARISON WITH THE GROUND-STATE OF (^{CF3}**L**^{1})$_2$FE AND THE HIGH-SPIN MODEL COMPLEX (^{CF3}**L**^{2})$_2$FE. 50

REFERENCES 51

EXPERIMENTAL DETAILS

Materials

($(^{CF3}\mathbf{L}^{1})_2$Fe and $(^{CF3}\mathbf{L}^{2})_2$Fe were synthesized following published protocols.[1,2] Unless otherwise specified, air-sensitive manipulations were carried either in a N_2 filled glove box or using standard Schlenk techniques under Argon. 2-acetylphenylboronic acid (Combi-Blocks), 2-formylphenylboronic acid (Combi-Blocks), 4-amino-3-bromo-5-nitrobenzotrifluoride (Combi-Blocks), hydrazine hydrate (Acros), formic acid (Fluka), 8-bromoquinoline (AK Scientific), $Pd(PPh_3)_4$ (Sigma Aldrich), $Pd_2(dba)_3$ (Sigma Aldrich), DPPF (Sigma Aldrich), Iron(II) trifluoromethanesulfonate ($Fe(OTf)_2$, Sigma Aldrich), and sodium *tert*-butoxide (NaO*t*Bu, Alfa Aesar) were purchased and used without further purification. Organic solvents (ACS grade) were dried and distilled using appropriate drying agents prior to use for both synthesis and solution characterization. The iron(II) complexes are prone to oxidation upon dissolution, hence samples were prepared and stored under an inert atmosphere. The samples for optical measurements were prepared in anhydrous toluene under a N_2 atmosphere using standard Schlenk line techniques.

Instrumentation and Methods

1- and 2D NMR spectra were recorded on Bruker Avance 300 MHz, Bruker Avance Neo 400 MHz, or Bruker Avance – III 500 MHz spectrometers. ^{1}H and ^{13}C{^{1}H} NMR spectra were referenced to residual solvent peaks.[3] High resolution mass spectra (HRMS) were recorded using a Bruker microOTOF-QIII. UV-Vis absorption spectra were collected on a Cary 5000 UV-Vis-NIR spectrophotometer using either 10 mm x 10 mm cuvettes (1 cm path length) or 0.1 mm x 10 mm cuvettes (0.1 cm path length). Spectroelectrochemical experiments were performed using an air-tight room temperature OTTLE cell from the University of Reading. A three-electrode system

of platinum minigrid working (32 wires/cm), auxiliary and Ag wire pseudo-reference electrodes was used.[4]

Optical Transient Absorption

Initial optical transient absorption spectroscopy experiments expanding the window of observation into the NIR region (820–1400 nm) were performed in the Lord Porter Ultrafast Laser Laboratory (ULS) at The University of Sheffield, using a Helios system (HE-VIS-NIR-3200) provided by Ultrafast Systems. A Ti:Sapphire regenerative amplifier (Spitfire ACE PA-40, Spectra-Physics) provides 800 nm pulses (40 fs FWHM, 10 kHz, 1.2 mJ). The 400 nm pump pulses (2.5 kHz, 0.2 μJ) were generated through frequency doubling of the amplifier fundamental. The pump was focused onto the sample to a beam diameter of ∼190 μm. The Near IR continuum (820–1400 nm) was generated using a 1 cm sapphire crystal and a portion of the amplifier fundamental. The intensity of the probe light transmitted through the sample was measured using an InGaAs sensor, with a resolution of 5 nm. Prior to the generation of the white light, the 800 nm pulses were passed through a computer-controlled optical delay line (DDS300, Thorlabs), which provides up to 8 ns of pump-probe delay. The instrument response function was approximated to be 100 fs (FWHM), based on the temporal duration of the coherent artifact signal from neat acetonitrile.

Broadband transient absorption spectra from 370 nm (3.4 eV) to 1200 nm (1 eV) were obtained by using an in-house build setup based on a Spitfire Pro XP (Spectra Physics) laser amplifier system that produces ~80 fs pulses at a central wavelength of 796 nm at 4 kHz repetition rate. A solution of $(^{\mathbf{CF3}}\mathbf{L^1})_2$Fe was prepared with absorption of ~0.4-0.6 at 598 nm in a 1 mm cuvette, translating to a concentration of ~0.3 mM. The laser output is split into two parts that are used to pump two collinear optical parametric amplifiers (TOPAS-C, Light Conversion). The first TOPAS is used to generate the pump beam at 600 nm. The second TOPAS is used to generate a

NIR beam (1350 nm) that is focused onto a 5 mm CaF_2 crystal to generate a supercontinuum probe beam. The delay between pump and probe beams is introduced by a computer-controlled delay stage (Aerotech) placed in the probe beam's path. After supercontinuum generation the probe pulses are split into two parts: the former overlapping with the pump pulse in the sample volume and the latter serving as a reference. The probe and the reference beams are then relayed onto the entrance slit of a prism spectrograph and dispersed onto a double photo-diode array, each holding 512 elements (Pascher Instruments). The power of excitation pulse was 2 mW. The diameters of the pump and the probe were ~600 µm and 150 µm, respectively. Excitation fluency $\sim 10^{15}$ photos/(cm^2 × pulse). The polarization between pump and probe beams was set to either perpendicular polarizations (90°) or magic angle (~54.7°) by placing a Berek compensator in the pump beam. The transient absorption spectra recorded at magic angle excitation avoid contributions from re-orientational dynamics but include a hole in the spectra around the excitation wavelength due to pump scattering. In contrast, perpendicular polarization affords the complete spectral coverage, but with the risk of contamination of pump-induced processes that lead to the change of the dipole moment orientation of the probed transitions. The instrument response function (IRF) is ~100 fs. The transient absorption data was recorded at room temperature in a quartz cuvette with a 0.1 mm beam path and two 0.1 mm thick windows to minimize contributions from coherent artefacts at the ultrafast timescales. The optical density was adjusted to ~0.3 at the excitation wavelength. The photostability of the samples was very high as measured steady-state absorption spectra before and after transient absorption experiments were identical. Background- and chirp-correction of the transient absorption data were performed in the software KiMoPack.[5]

SPECTROELECTROCHEMISTRY

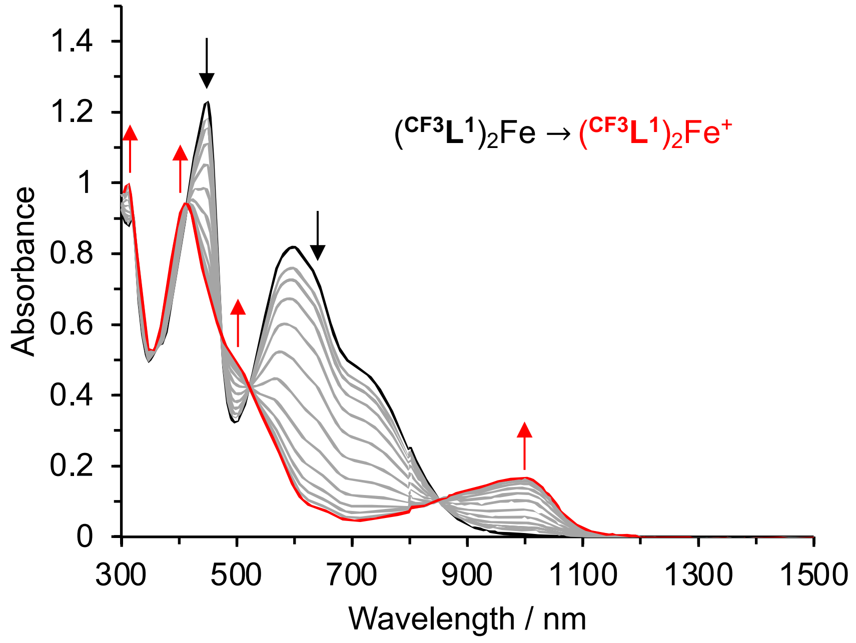

Figure S1. Spectroelectrochemically collected UV-vis-NIR absorption spectra of the oxidation of $(^{CF3}\mathbf{L}^{1})_2$Fe, with oxidative potentials applied from -0.20 V to 0.145 V vs. Ag wire.

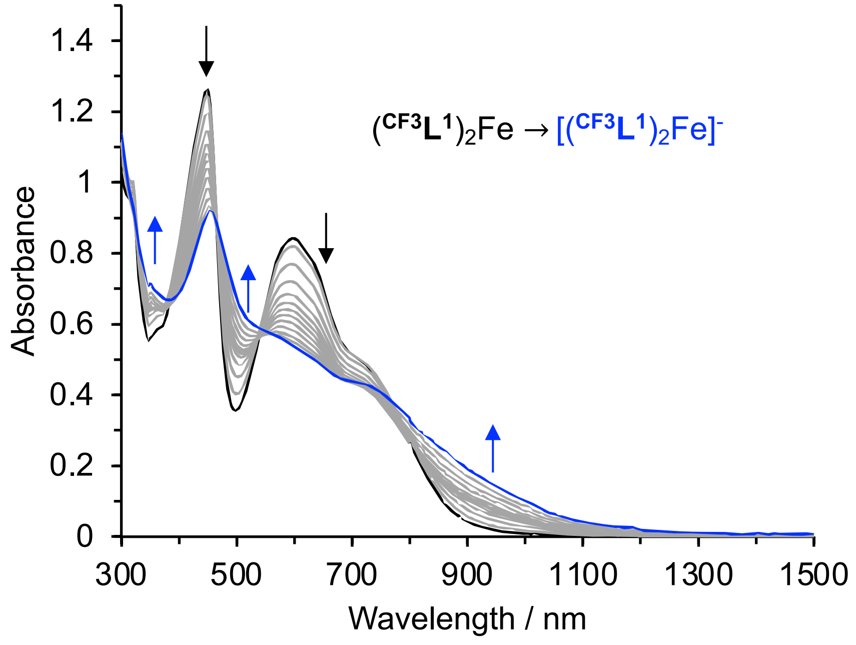

Figure S2. Spectroelectrochemically collected UV-vis-NIR absorption spectra of the reduction of $(^{CF3}\mathbf{L}^{1})_2$Fe, with reductive potentials applied from -0.70 V to -1.05 V vs. Ag wire.

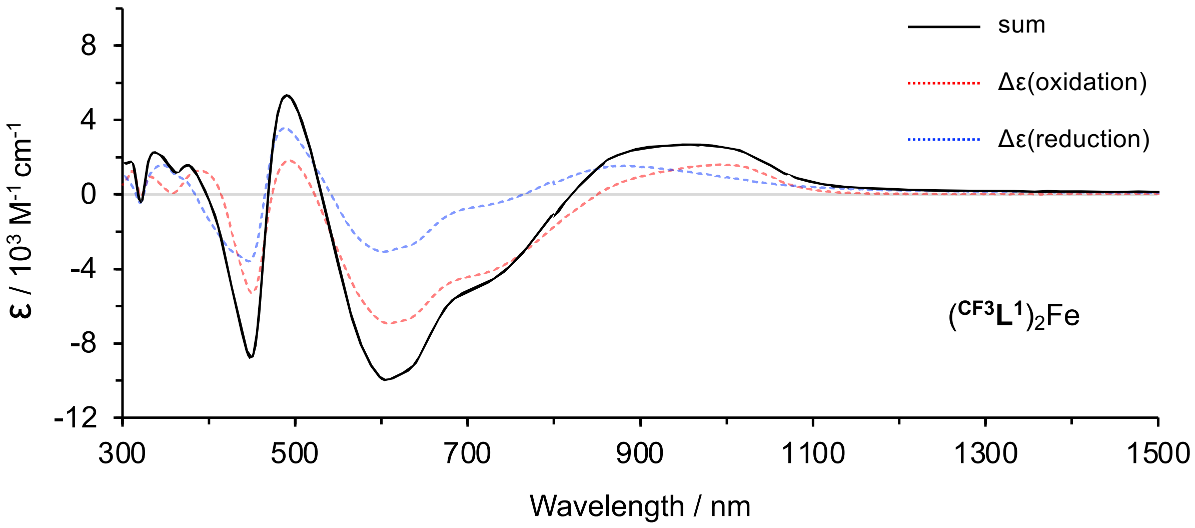

Figure S3. Calculated difference spectrum for the PALCT excited state of (^{CF3}**L**^{1})$_2$Fe (black solid trace), overlaid with the individual difference spectra for the oxidized complex (red dotted trace) and the reduced complex (blue dotted trace).

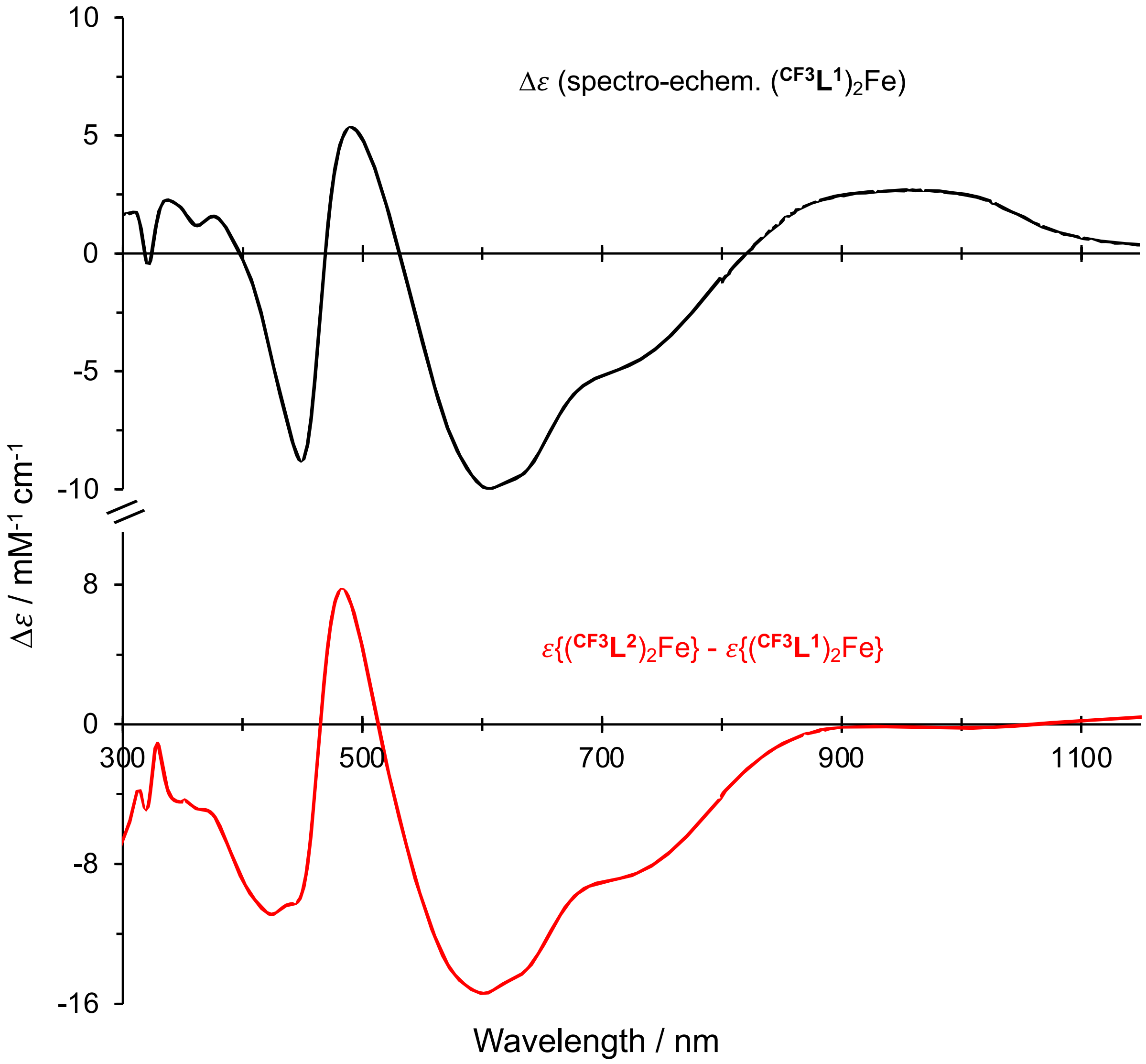

Figure S4. Simulated difference spectra for the PALCT excited state (top) and ^{5}MC excited state (bottom) for $(^{CF3}\mathbf{L^1})_2Fe$.

oTA SUPPORTING DISCUSSION AND FIGURES

Initial experiments carried out at Sheffield focused on changes in the NIR (820–1400 nm) on ultrafast (fs) to slower (ns) timescales, following low energy (780 nm) photoexcitation. These preliminary experiments revealed time-resolved features in this spectral region that prompted the wide-band experiments subsequently carried out at Lund. Specifically, in these initial experiments, for $(^{\mathbf{CF3}}\mathbf{L}^{\mathbf{1}})_2$Fe, short-lived features with a time constant of ~160 fs (which we could not rule out as the toluene solvent response) were observed, principally as sharp feature at 830 nm and bleach at 1100 (Figures S5a-b). Longer-lived features (~3.28 ns) comprised of a band at 900 and broad absorption between 920-1300 nm, were also evident. The observation of ESAs, in particular, in the NIR region raised the question as to whether the intensity of these features was commensurate or not with the strong ESA observed in the visible region.[1,6]

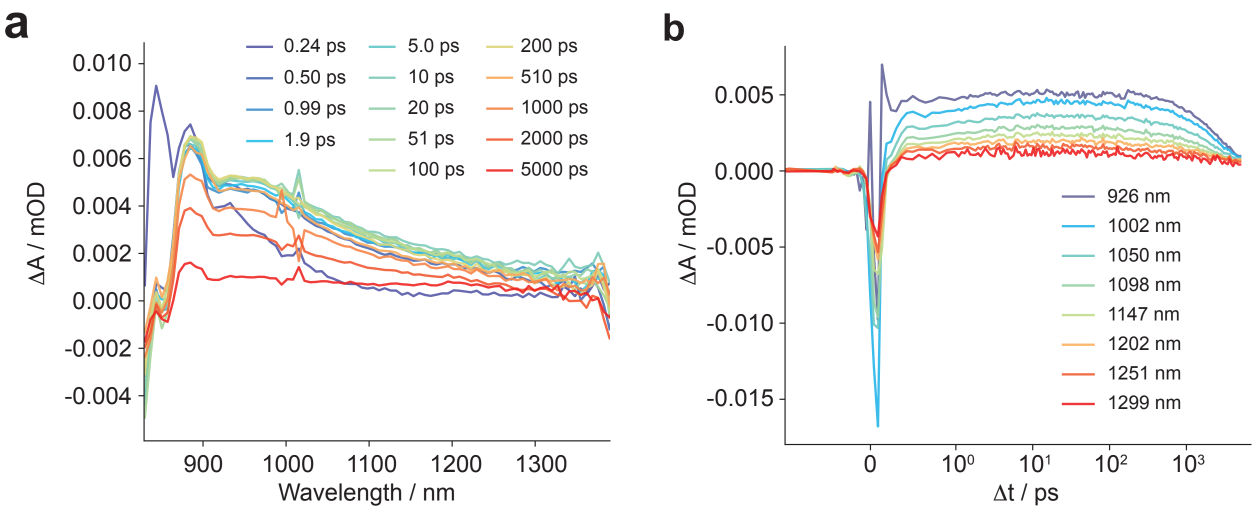

Figure S5. (a) NIR oTA spectra and (b) kinetic traces for $(^{\mathbf{CF3}}\mathbf{L}^{\mathbf{1}})_2$Fe ($\lambda_{excitation}$ = 780 nm).

For subsequent experiments carried out at Lund, the solvent response was found to be (substantially) weaker than the analyte signal in most of the oTA spectra collected (Figures S6-S7). For the kinetics of the very fast (<50 fs; Figure S7) component plotted at the same

wavelengths, solvent responses are weaker or much weaker and, in most cases, the shape of solvent response is quite different from the shape of the solute. We thus conclude that solvent artefacts do not complicate assignments of the solute dynamics.

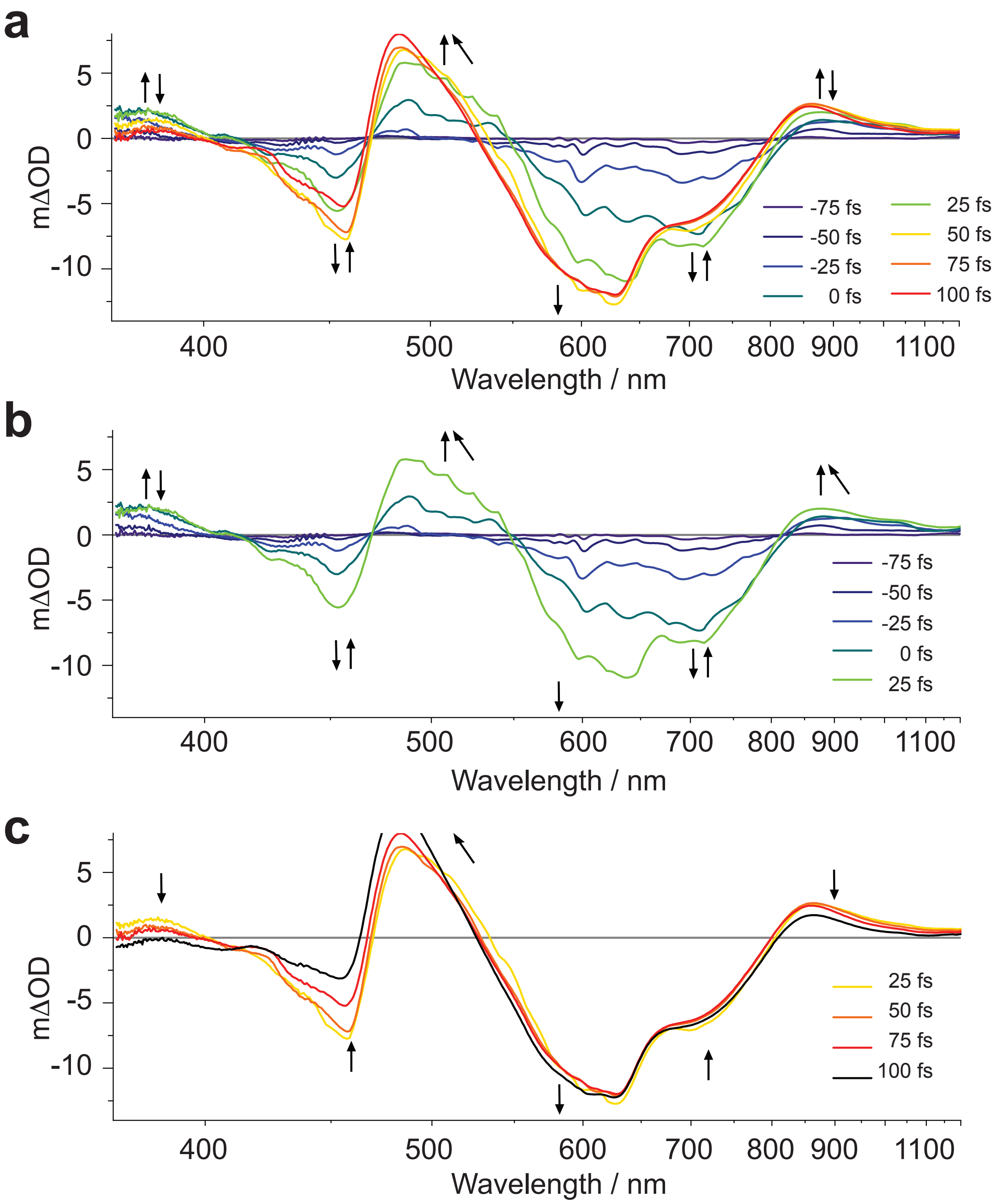

Figure S6. TA spectra of ($^{\mathbf{CF3}}\mathbf{L}^{\mathbf{1}}$)$_2$Fe in deaerated toluene at the ultrafast time scales, see delay times in inserts. (a) Two processes coincide at < 200 fs, observable by different dynamics for the various absorption features, which are highlighted by the arrows. The spectra dominated by the first and the second process are seen in (b) and (c), respectively. Based on our analysis, the first process is associated with ISC from the initially photogenerated ^{1}PALCT state to the ^{3}PALCT state and the second process is associated with depopulation of the ^{3}PALCT state to the MC manifold. See text for further discussion on the involvement of a ^{3}MC state in the deactivation pathway.

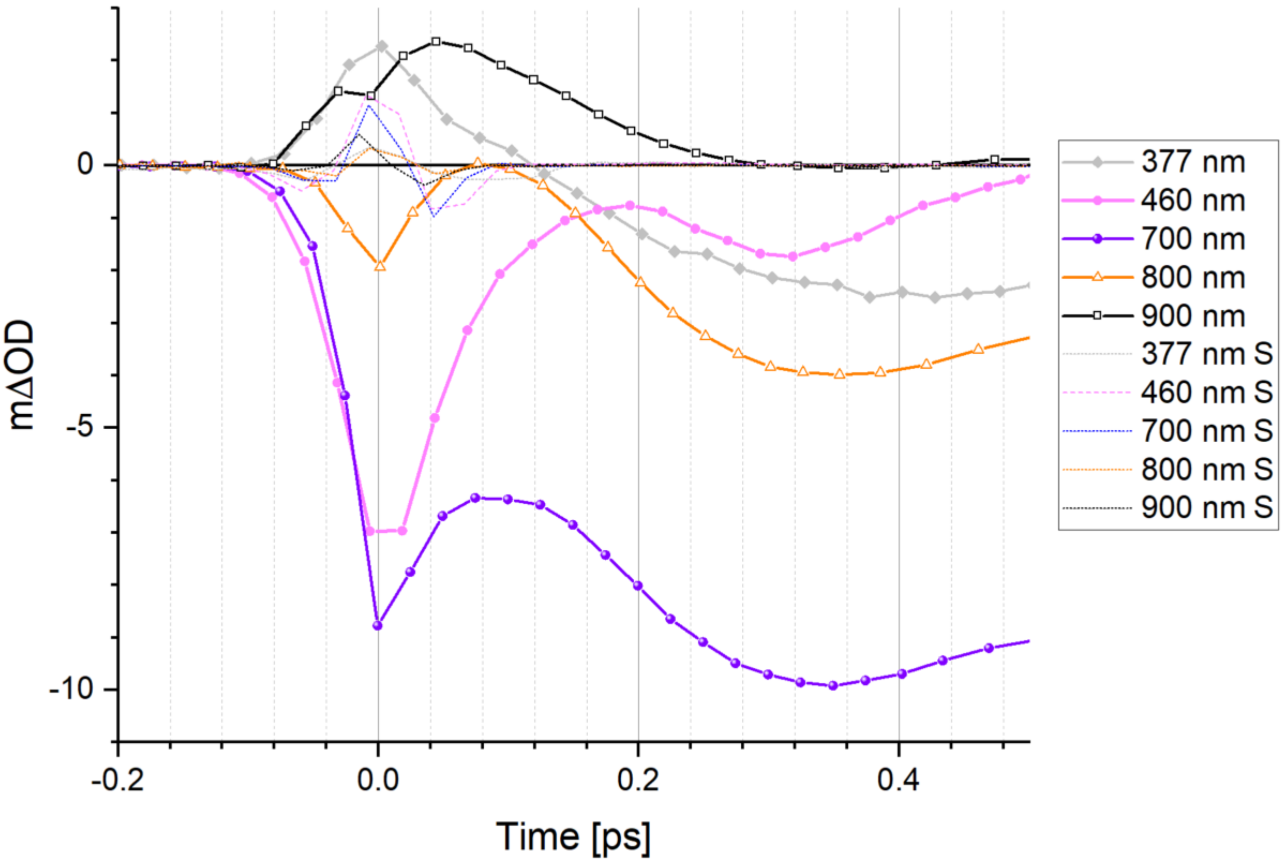

Figure S7. Measured kinetics at selected wavelengths (see insert) for (^{CF3}**L**^{1})$_2$Fe in deaerated toluene (symbols) and the corresponding solvent response (dashed lines, labelled "S" in legend).

OSCILLATIONS AND GLOBAL FITTING

Contribution of Oscillations to Fast Components

The strong oscillations noted in the main text (Figure 4a) complicate quantification of the dynamic spectral features and the rate components of the state-to-state transitions by fitting. While including oscillations in the fit function is conceptually appropriate, applying a global fitting procedure to the original data with sufficient variables and without any constrains resulted in models with nonsensical features and values. We note, however, that the outcomes of the fitting would vary depending on the initial inputs and can disagree with the qualitative analysis discussed above in terms of the number of state-to-state conversions and their decay times, as well as in the spectral shapes of the contributing components. In fact, there are several considerations which we find can justify neglecting the influence of the observed oscillations on excited state-to-state conversion. While these do create additional complications in the assignment of the early processes, as we point out in the main text of the article, the contribution of oscillations is relatively weak in some measured oTA spectral regions compared to changes associated with the PALCT-to-MC conversion, for example, and thus do not disturb the assignment. We therefore select these spectral regions to determine a relatively narrow range of variation of the rate of this conversion.

Another important consideration is the influence of oscillations on the ultrafast ^{1}PALCT$\rightarrow$^{3}PALCT transition. At these very fast timescales, we do not believe the spectral changes are significantly influenced by the observed oscillations for the following reasons. In the measured data, we observe that oscillations occur on a few hundreds of fs. Oscillations can either be excitation- or process-induced. If the oscillations are excitation-induced, then the change in the signal amplitude caused by oscillations is initially rather slow, due to the slowly changing amplitude of sine/cosine functions at zero delay. Instead, if oscillations are process-induced, they

can only appear after the fastest process is largely over. In either case, oscillations will have negligible contributions to the decay of the *fastest* time component.

Stepwise Global Fitting

In our global fitting, we opted to determine the starting parameter of the variables used in the fit in a stepwise fashion. In our procedure, we aim at searching for the global minimum and at defining initial guesses of all variables separately using the simplex algorithm, which is appropriate for searching a wide field of optimization. Eventually, we combined all fit functions together and used narrowly defined and limited initial guesses to fine-tune the variables in the global minimum by the least-mean-square algorithm. To quantify the spectral shapes of the early processes in terms of decay-associated spectra (DAS) and species-associated spectra (SAS), we first separated the contributions of the oscillations and the kinetic development to the measured spectra. We then fitted the kinetic development with a sequential kinetic model and the oscillations with a decaying phase-adjusted sinusoidal shape, requiring 5-6 kinetic components and 3-5 oscillatory components. With this large number of contributing components, we used the rigid approach outlined below to extract a meaningful analysis.

We used three different functions to model the oscillatory components. The oscillations were in each case treated as a separate "species" and started with the IRF and follow a dampened Sinus function, a dampened cosine function or a dampened cosine function that has an additional rise term (delayed initiation). This delayed rise represents a stretching of the IRF for all oscillations in the fit. In the combined fits the maximum of this rise was chosen to be 250 fs, as at this time we are sure that oscillations are present in the data.

All analysis steps were performed using the KiMoPack software package.[5] The Jupyter notebook containing the procedure, together with the raw data, is provided as additional Supporting Information. The step-by-step procedure of the quantification of oTA data is as follows:

1. The data were loaded and chirp-corrected to determine delay time zero for different probe wavelengths. This chirp correction was carefully refined twice during the analysis as part of the fitting. The Jupyter file contains the initial and the final chirp correction. A standard SVD analysis is part of the pre-fitting analysis procedure and used to estimate the expected numbers of contributing processes/spectra.
2. We then conducted preliminary fit of the kinetic decays over the entire measured spectral range on the time scale when oscillations have been completely dumped (more than 5 ps delays in this case). This fit used a simplex algorithm and were verified to result in a true global optima by checking a wide series of starting parameters using the AMPGO algorithm.7 The AMPGO performs many iterative simplex optimizations sampling a wide range of starting parameters and selecting the best of these iterations.
3. For the obtained decays with frozen parameters and spectra, the resulting decay function was extended towards zero time and convoluted with the IRF; then the convoluted TA signal generated by the above fit was subtracted from the measured data.
4. The residuals were then fitted using an oscillatory model (decaying single frequency oscillation) on the time scale outside IRF to avoid contributions from ISC and any potential solvent artefacts.
5. We then extended the fitted oscillation with frozen parameters and spectra towards zero time, convoluted it with IRF, and subtracted the generated TA signal from the residuals obtained from the previous step.

6. We next subtracted the first oscillation from the as-measured data and refined the kinetic development using the same starting parameters as before.

7. We subsequently evaluated the new residuals for presence of further oscillations and repeated steps 4-6 twice more for a total of three fitted oscillations. At this point, no clear oscillations were visible in the residuals.

8. After contributions of all three oscillations were removed from measured data, we were able to lock on six distinct decay times in the kinetics using a least-squares minimizer. This includes a process that is clearly visible as an isosbestic point in the raw data around 160-250 fs. This spectrum was evaluated and the errors extracted. This fit resulted in an R^2 value of 0.9991 and a sum of the squared errors of 1.72×10^{-3} /1.8×10^{-3} /1.71×10^{-3} if a (respectively) sinus / cosine / delayed cosine function was used. When using a simplex refinement algorithm, the model does not lock on the species at 250 fs that represents the 'isosbestic point' process in the least-squares minimizer modelling of the data. The results at this stage are shown in Tables S1, S4 and S7 and Figures S8, S13 and S18. In addition, see kinetic data in Figures S11a, S16a and S21a, and SAS in Figures S12a, S17a and S22a.

9. In the next step, we combined all three oscillations into a single model, freezing their frequencies and oscillation decay times but allowing the kinetic times to be optimized. In this fit, we calculated the spectra of both the oscillating and kinetic species simultaneously. This approach yielded a slightly better R^2 of 0.9995/0.9995/0.99936 and a sum of the squared errors of 9.8×10^{-4} /9.2×10^{-4} /1.22×10^{-3} if a (respectively) sinus, cosine, delayed cosine function was used. This reflects a slightly better fit since the spectra assigned to the oscillations were simultaneously refined. The results at this stage are shown in Tables S2, S5 and S8, and Figures S9, S14 and S19. See also kinetic data in Figures S11b, S16b and S21b and SAS in Figures S12b, S17b and S22b.

10. Finally, we permitted the oscillation and exponential decay functions to be refined in a very narrow range and performed a free optimization of all kinetic and oscillatory parameters. This yielded an R^2 of 0.9996/0.99966/0.99937 and a sum of the squared errors of $8.3x10^{-4}$/$6.5x10^{-4}$/$1.206x10^{-3}$ if a (respectively) sinus, cosine, delayed cosine function was used. This fit is also the only one that has no visible oscillating structures left in the fit residuals. The results at this stage are shown in Tables S3, S6 and S9, and Figures S10, S15, S20 and S16. See also kinetic data in Figures S11c, S16c and S21c and SAS in Figures S12c, S17c and S22c. We also removed the calculated oscillations from both the data and model, shown as the resulting pure kinetic plots in Figures S26.

Fitting Oscillations as a Sinus Function

Table S1. Parameters obtained for Model A (best $R^2 = 0.9991$). Separately fitted oscillations were subtracted and standard kinetics fitted spectra extracted.

**Oscillation 1**	550 fs period	735fs decay
**Oscillation 2**	300 fs period	844 fs decay
**Oscillation 3**	408 fs period	425 fs decay
K1	$30\ fs_{32\ fs}^{28\ fs}$	
K2	$100\ fs_{95\ fs}^{105\ fs}$	
K3	$250\ fs_{238\ fs}^{263\ fs}$	
K4	$12.28\ ps_{11.7\ ps}^{12.9\ ps}$	
K5	$131.7\ ps_{125\ ps}^{139\ ps}$	
K6	$3.5\ ns_{3.3\ ns}^{3.7\ ns}$	

Table S2. Parameters obtained for Model B (best $R^2 = 0.99948$). Frozen oscillations parameter and all spectra extracted.

**Oscillation 1**	550 fs period	735fs decay
**Oscillation 2**	300 fs period	844 fs decay
**Oscillation 3**	408 fs period	425 fs decay
K1	$4.7\ fs_{4.5\ fs}^{5\ fs}$	
K2	$100\ fs_{95\ fs}^{105\ fs}$	
K3	$250\ fs_{238\ fs}^{263\ fs}$	
K4	$15.9\ ps_{15.1\ ps}^{16.7\ ps}$	
K5	$127\ ps_{121\ ps}^{134\ ps}$	
K6	$3.52\ ns_{3.35\ ns}^{3.7\ ns}$	

Table S3. Parameters obtained for Model C (Best $R^2 = 0.99956$). All parameters and spectra were fitted.

**Oscillation 1**	$546\ fs_{519\ fs}^{574\ fs}$ period	$134\ fs_{127\ fs}^{140\ fs}$ decay
**Oscillation 2**	$300\ fs_{285\ fs}^{315\ fs}$ period	$513\ fs_{488\ fs}^{540\ fs}$ decay
**Oscillation 3**	$402\ fs_{382\ fs}^{422\ fs}$ period	$345\ fs_{328\ fs}^{363\ fs}$ decay
K1	$30\ fs_{28.5\ fs}^{31.5\ fs}$	
K2	$100\ fs_{95\ fs}^{105\ fs}$	
K3	$250\ fs_{238\ fs}^{263\ fs}$	
K4	$10.9\ ps_{10.4\ ps}^{11.5\ ps}$	
K5	$138.6\ ps_{132\ ps}^{145.9\ ps}$	
K6	$3.5\ ns_{3.3\ ns}^{3.7\ ns}$	

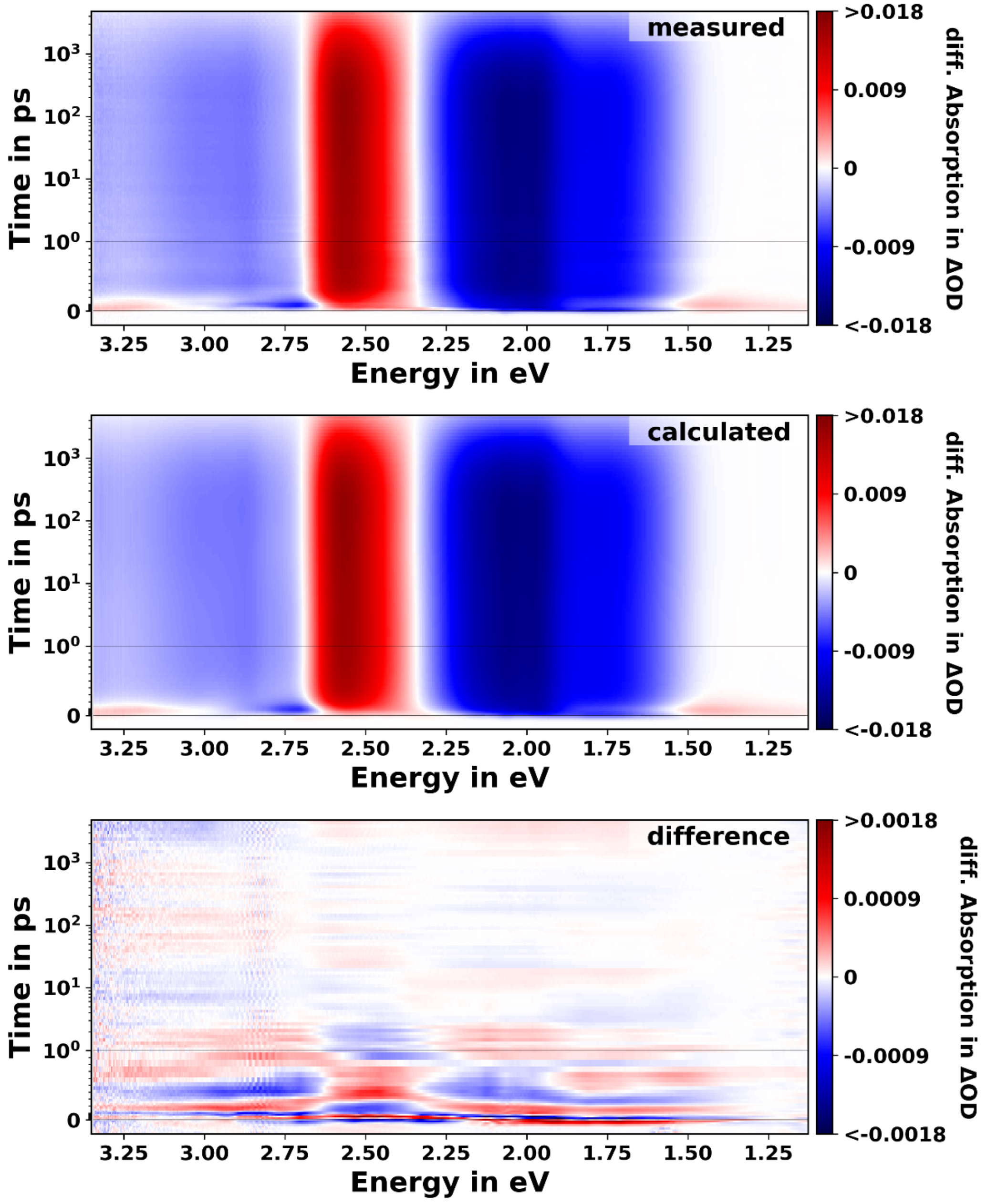

Figure S8. Measured, modelled and 10x the difference shown as two-dimensional matrices. Iterative fitting step as described in Step 8 in the procedure above using a sinus function for modelling the oscillations. Note: for this fit, the oscillations were removed from the data and model prior to fitting.

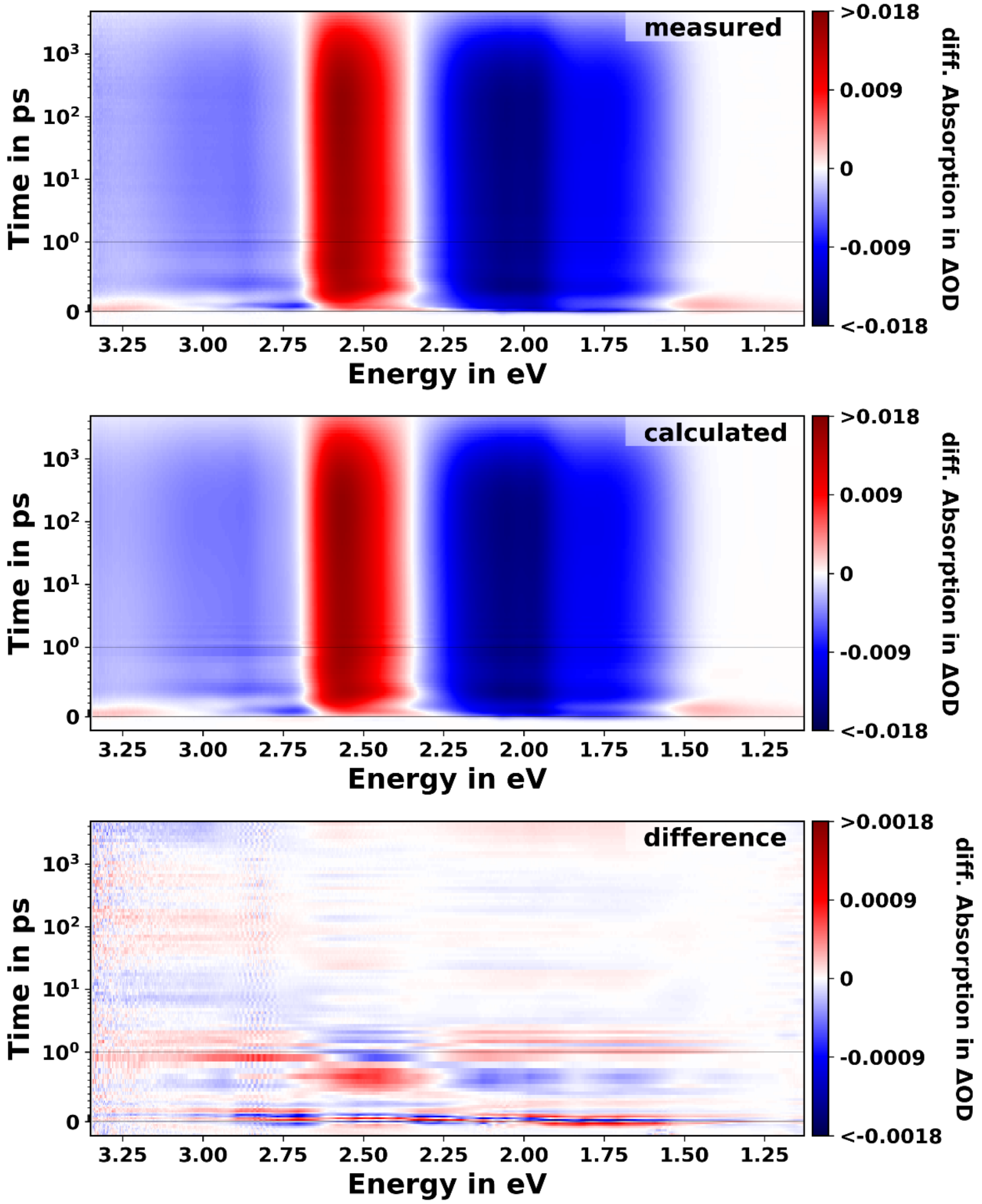

Figure S9. Measured, modelled, and 10x the difference shown as two-dimensional matrices. Fitting with oscillatory temporal parameters fixed, but spectra allowed to vary as described in step 9 in the procedure above using a sinus function for modelling the oscillations.

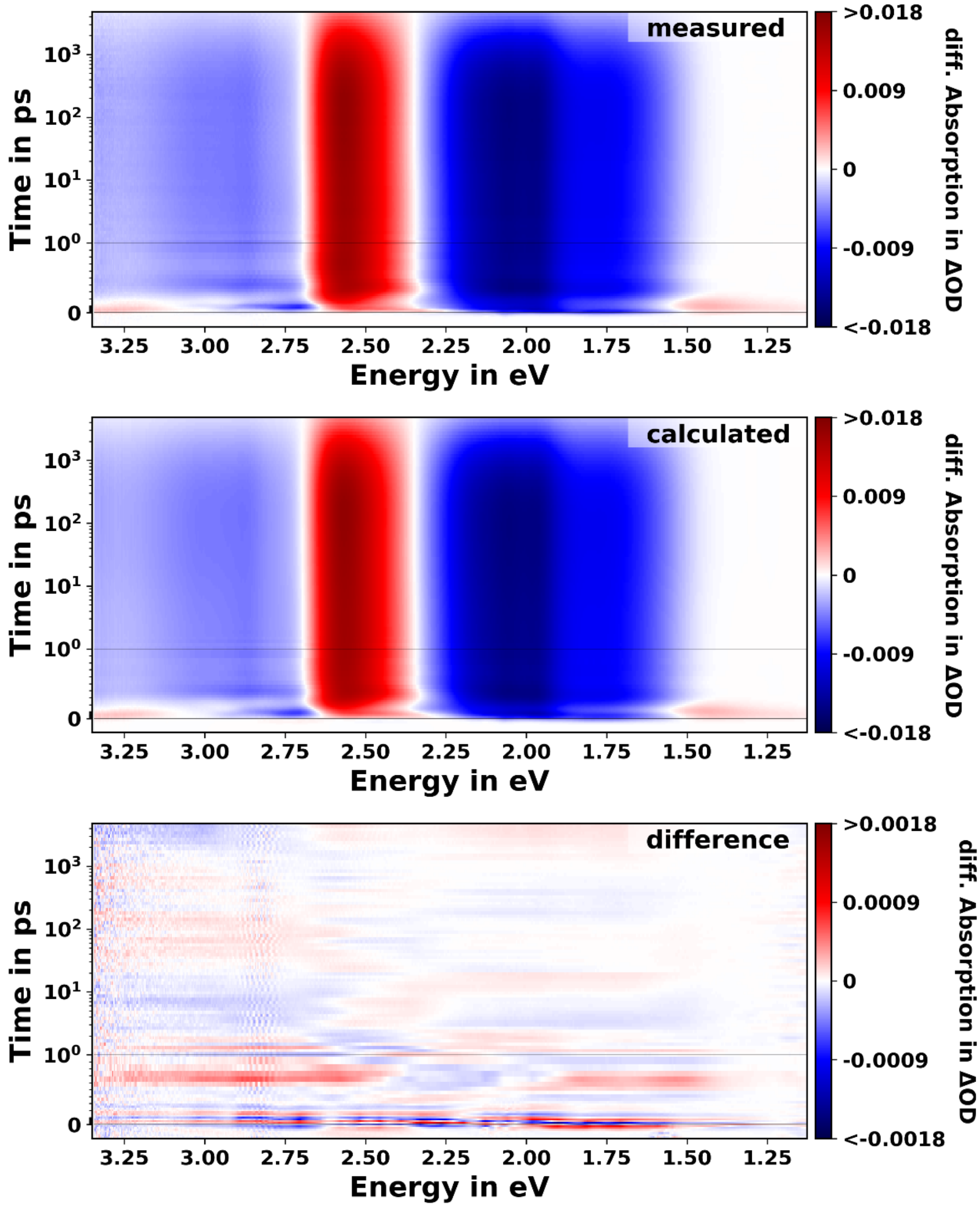

Figure S10. Measured, modelled, and 10x the difference shown as two-dimensional matrices. Fitting with all parameters allowed to vary as described in step 10 in the procedure above using a sinus function for modelling the oscillations.

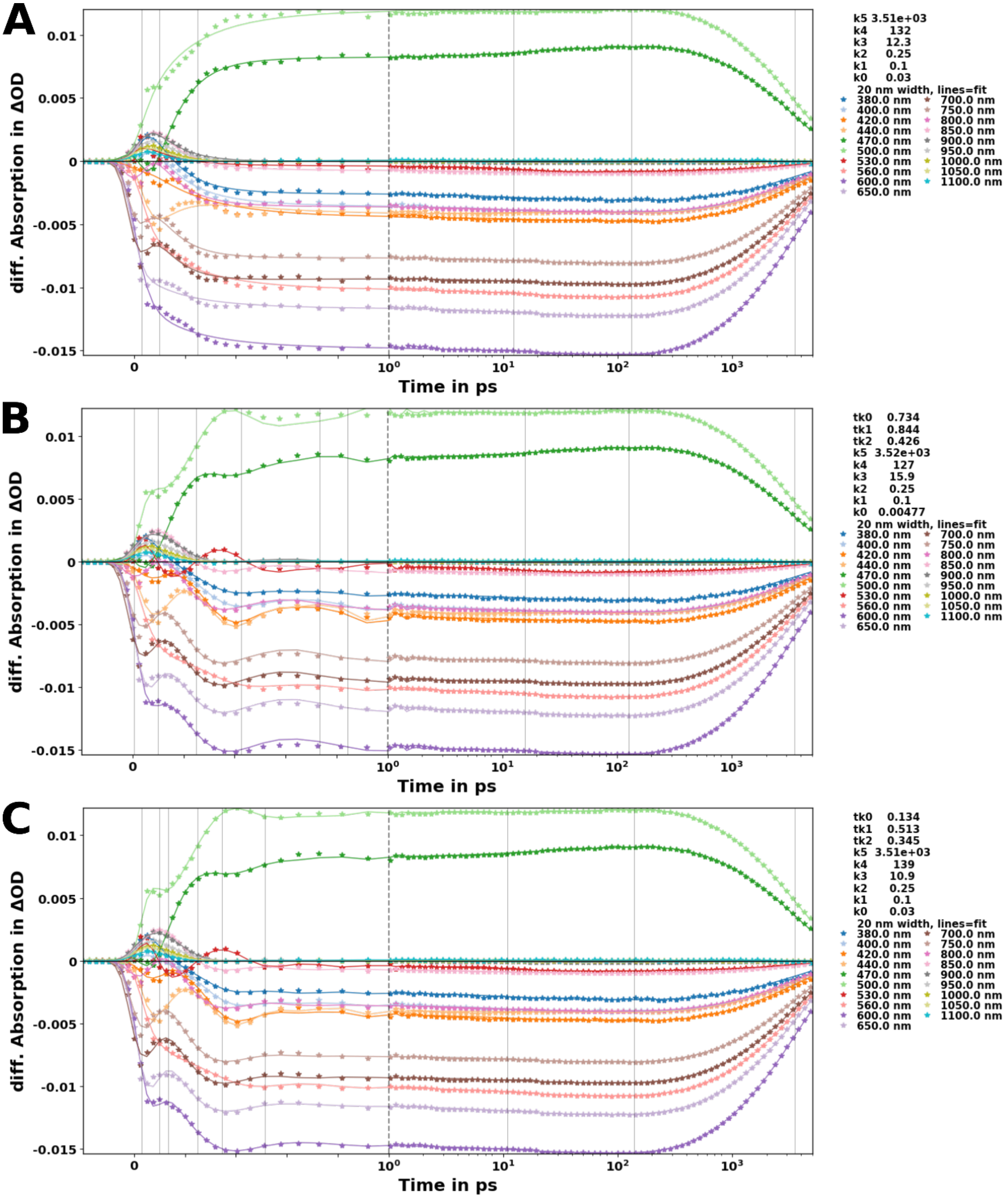

Figure S11. Kinetic development of the data for (^{CF3}**L**^{1})$_2$Fe. Dots are measured and chirp corrected data points. The lines originate from (a) Step 8 in the description above; (b) and (c) Steps 9 and 10 in the description above. In this model, the oscillations were modeled using a sinus function.

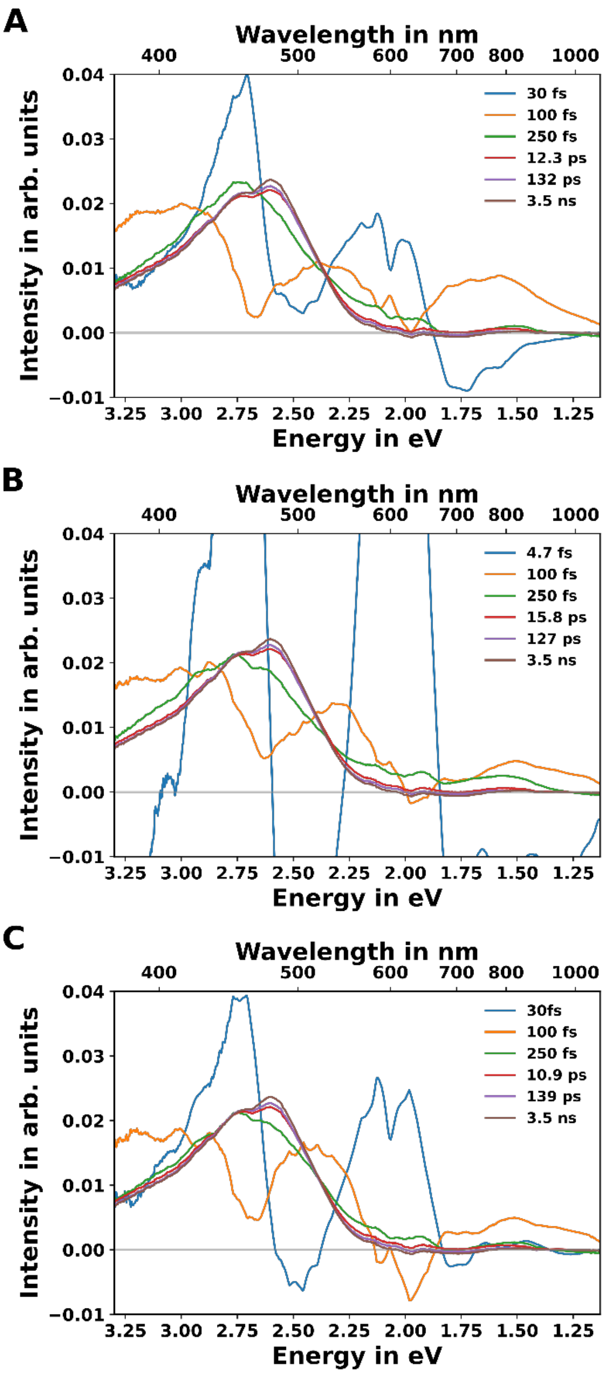

Figure S12. Extracted species-associated spectra from the fits for $(^{CF3}L^{1})_2Fe$ using a sinus function. (a) Kinetic modelling in Step 8 with removed oscillations and least-square optimizer. (b) Kinetic and spectral modelling in step 9 with locked oscillation temporal parameter. (c) Freely refining temporal and oscillator parameters. From the extracted SAS spectra, the ground state measured absorption spectra were subtracted, after it was scaled to the average intensity of the 10-15ps feature between 600 and 700nm.

Fitting Oscillations as a Cosine Function

Table S4. Parameters obtained for Model A (best $R^2 = 0.9991$). Separately fitted oscillation were subtracted and standard kinetic fitted spectra extracted.

**Oscillation 1**	430 fs period	371 fs decay
**Oscillation 2**	710 fs period	888 fs decay
**Oscillation 3**	738 fs period	232 fs decay
K1	$30\ fs_{32\ fs}^{28\ fs}$	
K2	$100\ fs_{95\ fs}^{105\ fs}$	
K3	$250\ fs_{238\ fs}^{263\ fs}$	
K4	$12.1\ ps_{11.5\ ps}^{12.8\ ps}$	
K5	$132\ ps_{125\ ps}^{139\ ps}$	
K6	$3.5\ ns_{3.3\ ns}^{3.7\ ns}$	

Table S5. Parameters obtained for Model B (best $R^2 = 0.99949$). Frozen oscillation parameter, all spectra extracted.

**Oscillation 1**	430 fs period	371 fs decay
**Oscillation 2**	710 fs period	888 fs decay
**Oscillation 3**	738 fs period	232 fs decay
K1	$29\ fs_{31\ fs}^{28\ fs}$	
K2	$100\ fs_{95\ fs}^{105\ fs}$	
K3	$249\ fs_{237\ fs}^{262\ fs}$	
K4	$12.5\ ps_{11.9\ ps}^{13.2\ ps}$	
K5	$130\ ps_{124\ ps}^{138\ ps}$	
K6	$3.5\ ns_{3.3\ ns}^{3.7\ ns}$	

Table S6. Parameters obtained for Model C (best $R^2 = 0.99956$). All parameters and all spectra fitted.

**Oscillation 1**	$441\ fs_{418\ fs}^{463\ fs}$ period	$121\ fs_{115\ fs}^{128\ fs}$ decay
**Oscillation 2**	$725\ fs_{689\ fs}^{761\ fs}$ period	$194\ fs_{185\ fs}^{205\ fs}$ decay
**Oscillation 3**	$730\ fs_{693\ fs}^{766\ fs}$ period	$128\ fs_{122\ fs}^{135\ fs}$ decay
K1	$20\ fs_{19\ fs}^{21\ fs}$	
K2	$96\ fs_{92\ fs}^{102\ fs}$	
K3	$193\ fs_{183\ fs}^{203\ fs}$	
K4	$9.1\ ps_{8.6\ ps}^{9.6\ ps}$	
K5	$135\ ps_{128\ ps}^{142\ ps}$	
K6	$3.5\ ns_{3.3\ ns}^{3.7\ ns}$	

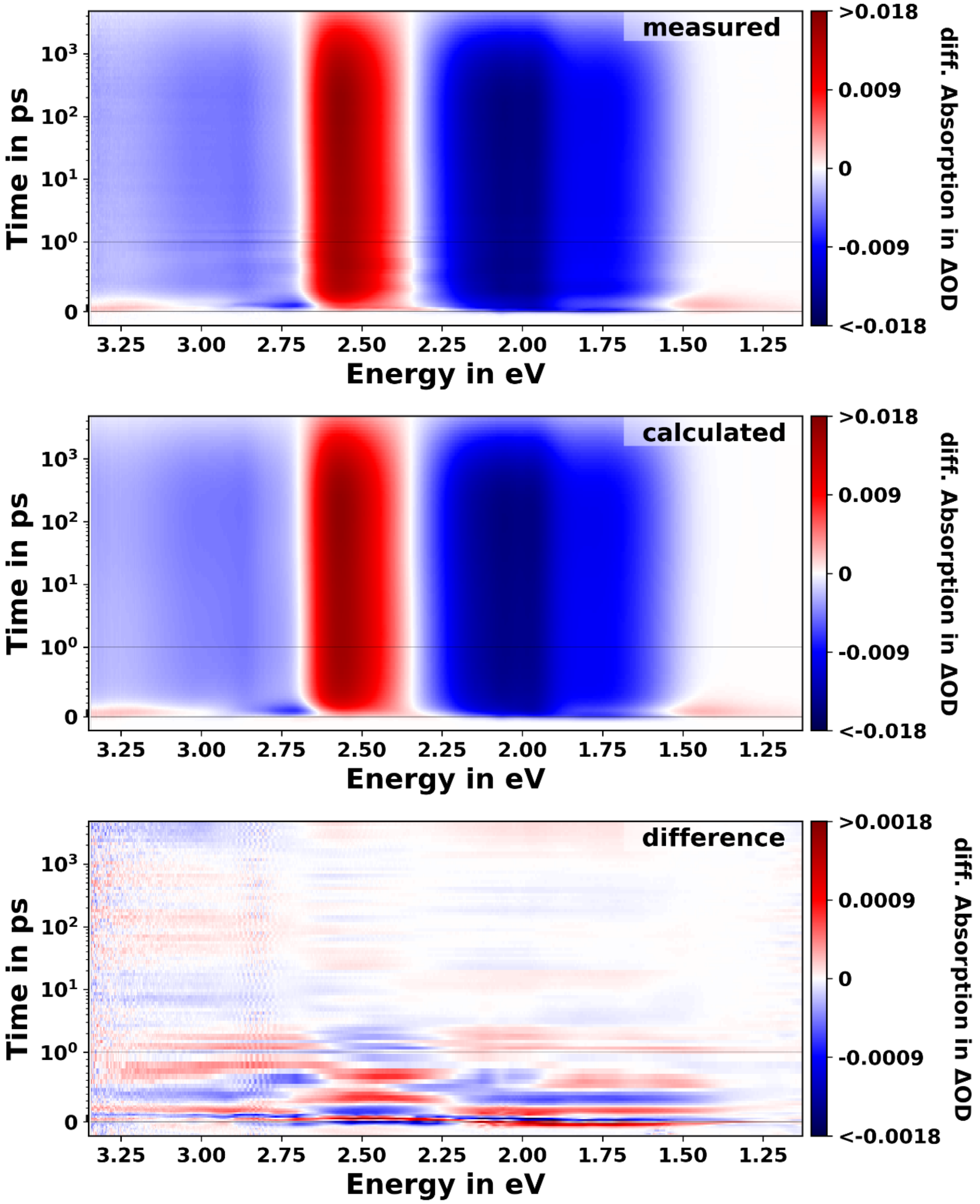

Figure S13. Measured, modelled, and 10x the difference shown as two-dimensional matrices. Iterative fitting step as described in Step 8 in the procedure above using a cosine function for modelling the oscillations. Note that for this fit the oscillations were removed from the data and model prior to fitting.

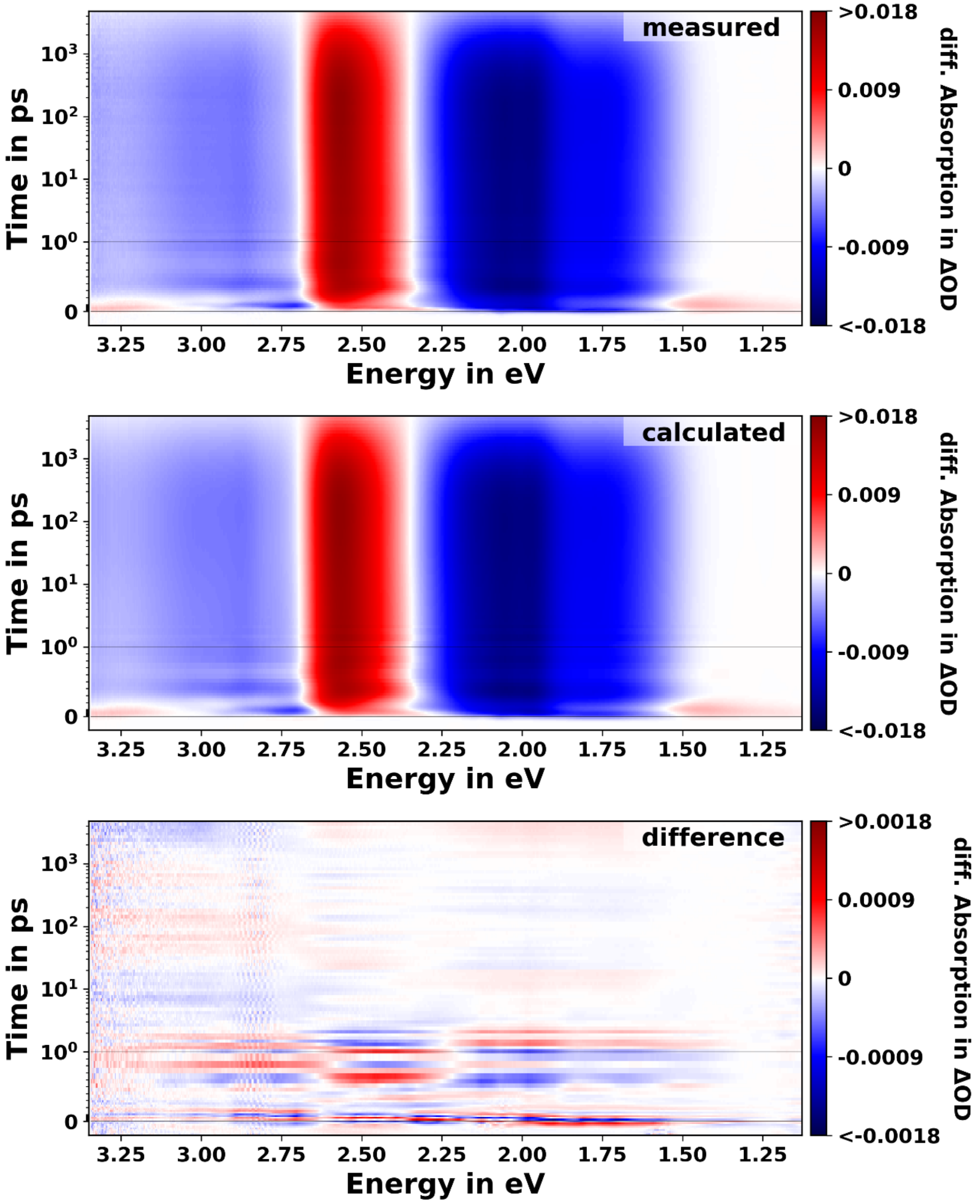

Figure S14. Measured, modelled, and 10x the difference shown as two-dimensional matrices. Fitting with oscillatory temporal parameters fixed, but spectra allowed to vary as described in step 9 in the procedure above using a cosine function for modelling the oscillations.

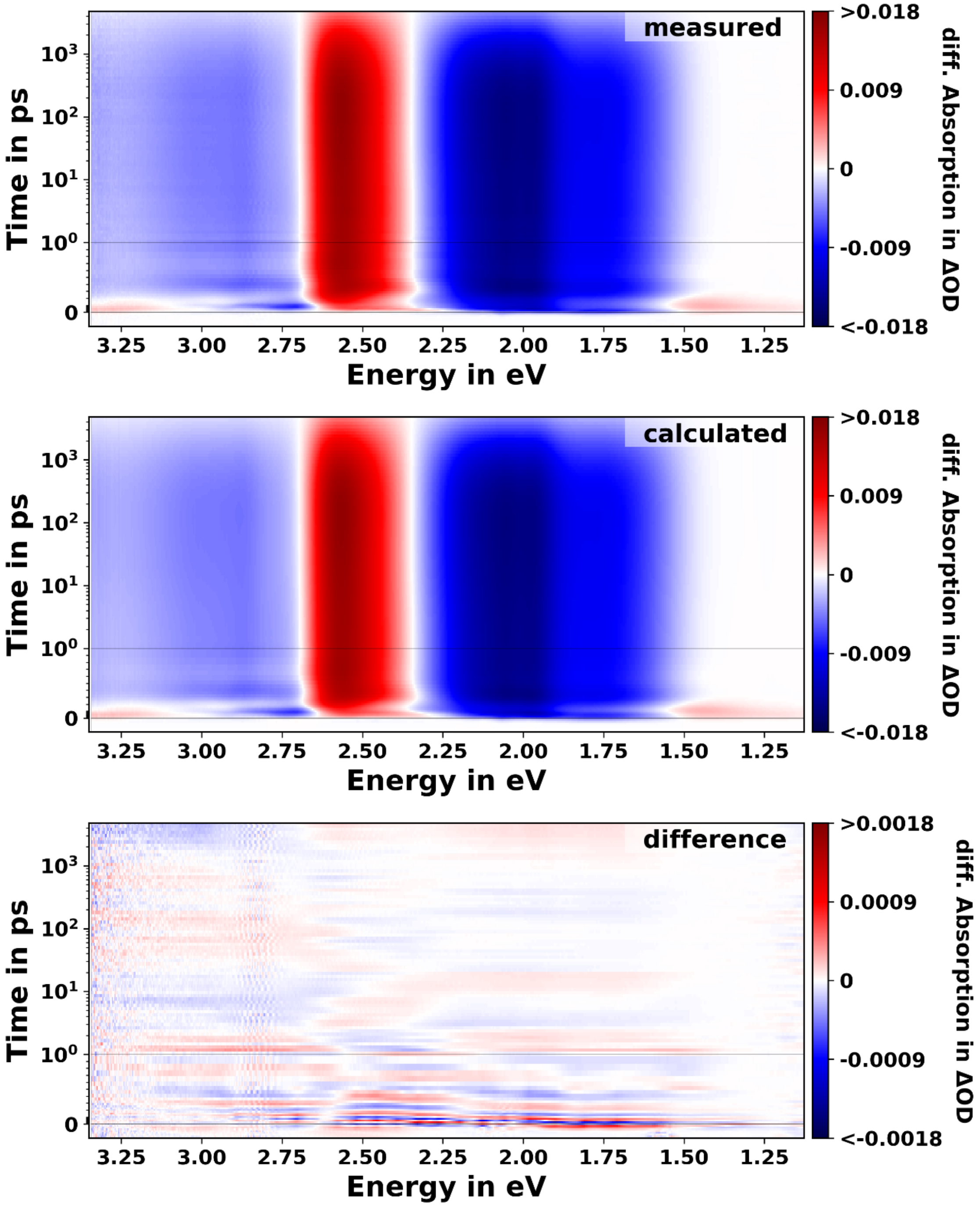

Figure S15. Measured, modelled, and 10x the difference shown as two-dimensional matrices. Fitting with all parameters allowed to vary as described in Step 10 in the procedure above using a cosine function for modelling the oscillations.

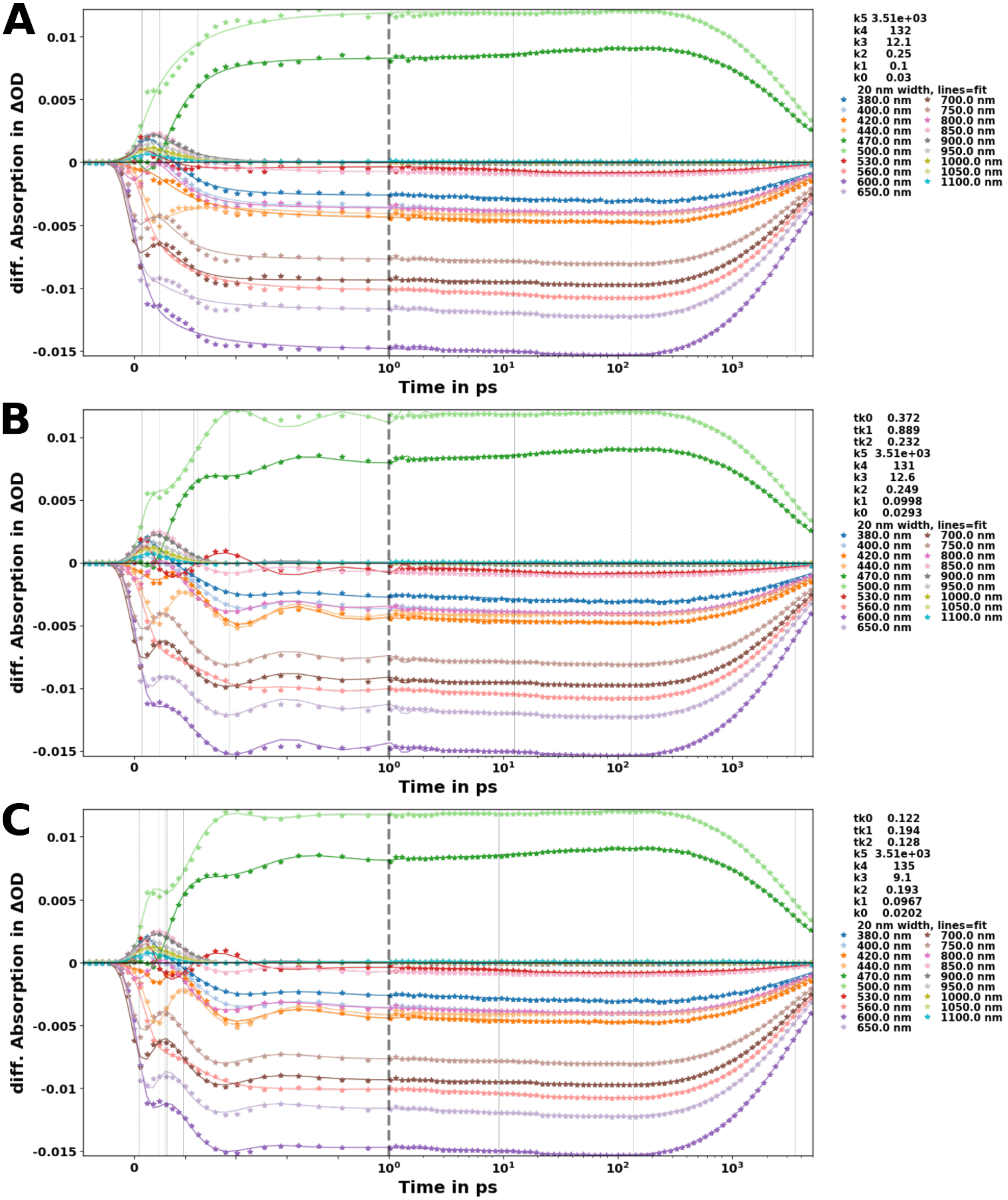

Figure S16. Kinetic development of the data for $(^{CF3}L^1)_2Fe$. Dots are measured and chirp corrected datapoints. The lines originate from (a) Step 8 in the description above; (b) and (c) Steps 9 and 10 in the description above. In this model, the oscillations were modeled using a cosine function.

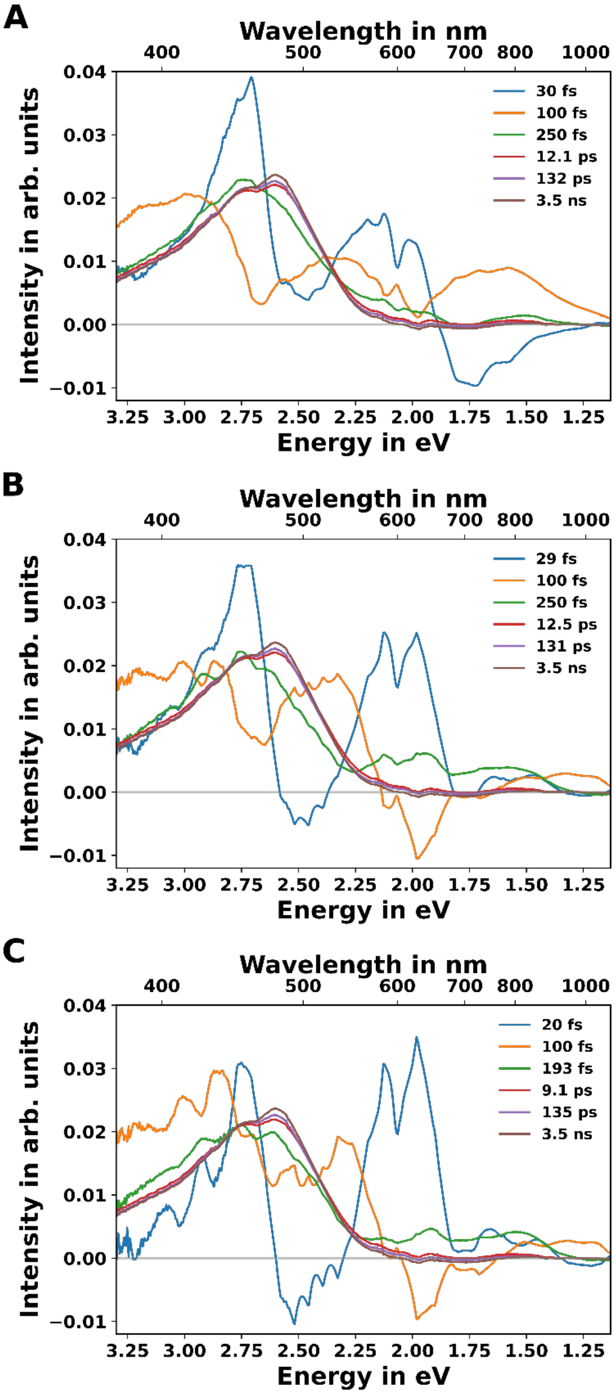

Figure S17. Extracted species-associated spectra from the fits for $(^{CF3}\mathbf{L}^{1})_2Fe$ using a cosine function. (a) Kinetic modelling in Step 8 with removed oscillations and least-square optimizer. (b) Kinetic and spectral modelling in Step 9 with locked oscillation temporal parameter. (c) Freely refining temporal and oscillator parameters. From the extracted SAS spectra, the ground state measured absorption spectra were subtracted, after it was scaled to the average intensity of the 10-15 ps feature between 600 and 700nm.

Fitting Oscillations as a Cosine Function with a Delayed Rise

Table S7. Parameters obtained for Model A (best R^2 = 0.9991). Separately fitted oscillations were subtracted and standard kinetic fitted spectra extracted.

**Oscillation 1**	$400\ fs$ period	$155\ fs$ decay	326 fs rise
**Oscillation 2**	$718\ fs$ period	$470\ fs$ decay	151 fs rise
**Oscillation 3**	$509\ fs$ period	$834\ fs$ decay	276 fs rise
K1	$30\ fs_{32\ fs}^{28\ fs}$		
K2	$100\ fs_{95\ fs}^{105\ fs}$		
K3	$250\ fs_{238\ fs}^{263\ fs}$		
K4	$12.3\ ps_{11.7\ ps}^{13\ ps}$		
K5	$132\ ps_{125\ ps}^{139\ ps}$		
K6	$3.51\ ns_{3.3\ ns}^{3.7\ ns}$		

Table S8. Parameters obtained for Model B (best R^2 = 0.99936). Frozen oscillation parameter, all spectra extracted.

**Oscillation 1**	$400\ fs$ period	$155\ fs$ decay	$158\ fs_{154\ fs}^{162\ fs}$ rise
**Oscillation 2**	$718\ fs$ period	$470\ fs$ decay	$158\ fs_{154\ fs}^{162\ fs}$ rise
**Oscillation 3**	$509\ fs$ period	$834\ fs$ decay	$158\ fs_{154\ fs}^{162\ fs}$ rise
K1	$30\ fs_{32\ fs}^{28\ fs}$		
K2	$100\ fs_{95\ fs}^{105\ fs}$		
K3	$249\ fs_{237\ fs}^{262\ fs}$		
K4	$12.5\ ps_{11.9\ ps}^{13.2\ ps}$		
K5	$135\ ps_{128\ ps}^{142\ ps}$		
K6	$3.51\ ns_{3.3\ ns}^{3.7\ ns}$		

Table S9. Parameters obtained for Model C (best $R^2 = 0.99937$). All parameters and all spectra fitted.

**Oscillation 1**	$390\,fs_{370\,fs}^{410\,fs}$ period	$154\,fs_{146\,fs}^{162\,fs}$ decay	$158\,fs_{154\,fs}^{162\,fs}$	rise
**Oscillation 2**	$721\,fs_{685\,fs}^{757\,fs}$ period	$467\,fs_{445\,fs}^{492\,fs}$ decay	$158\,fs_{154\,fs}^{162\,fs}$	rise
**Oscillation 3**	$505\,fs_{480\,fs}^{530\,fs}$ period	$830\,fs_{793\,fs}^{877\,fs}$ decay	$158\,fs_{154\,fs}^{162\,fs}$	rise
K1	$30\,fs_{28\,fs}^{32\,fs}$			
K2	$100\,fs_{95\,fs}^{105\,fs}$			
K3	$249\,fs_{234\,fs}^{262\,fs}$			
K4	$12.1\,ps_{11.53\,ps}^{12.7\,ps}$			
K5	$121\,ps_{115\,ps}^{127\,ps}$			
K6	$3.5\,ns_{3.3\,ns}^{3.7\,ns}$			

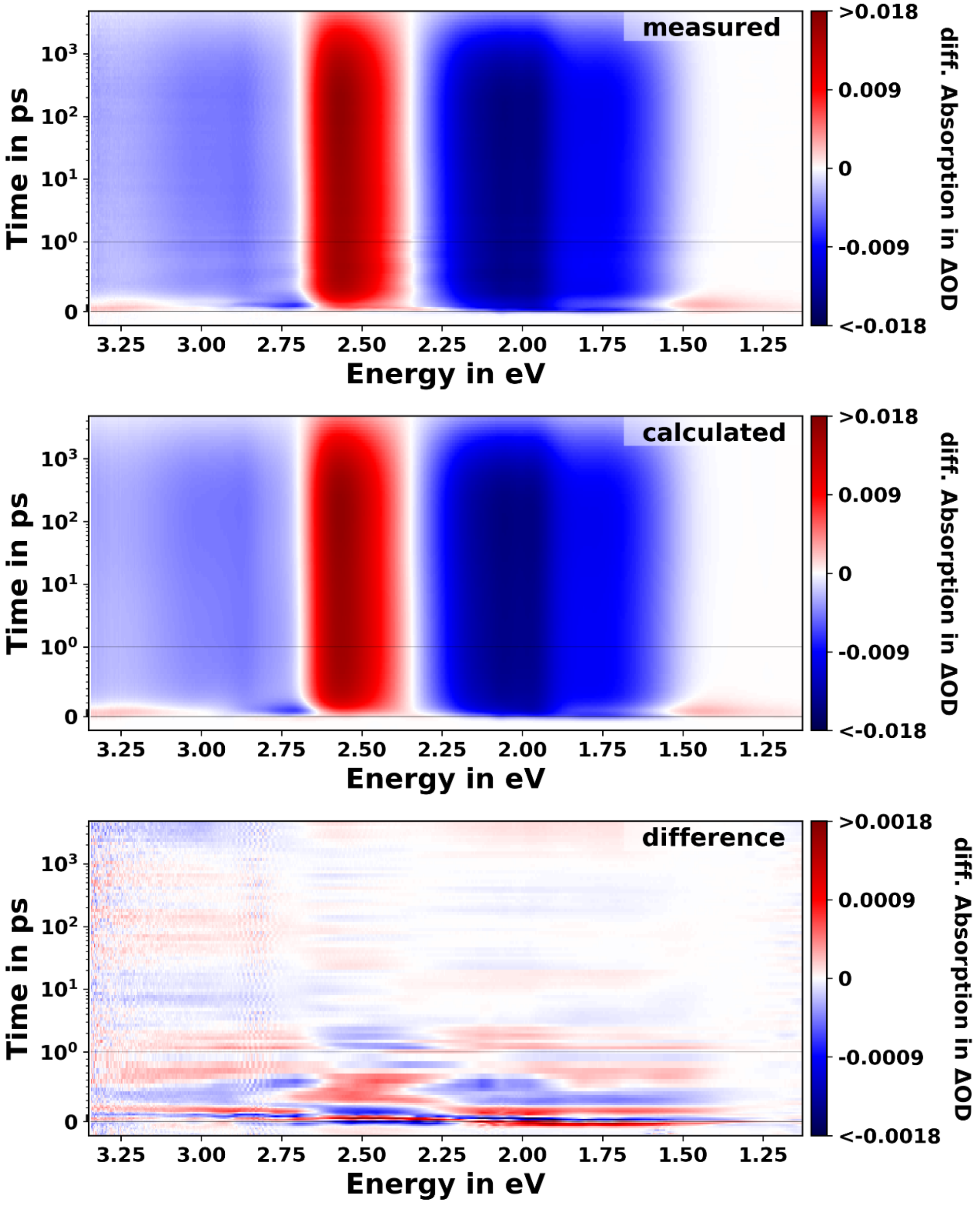

Figure S18. Measured, modelled, and 10x the difference shown as two-dimensional matrices. Iterative fitting step as described in step 8 in the procedure above using a cosine function for modelling the oscillations. Note that for this fit the oscillations were removed from the data and model prior to fitting.

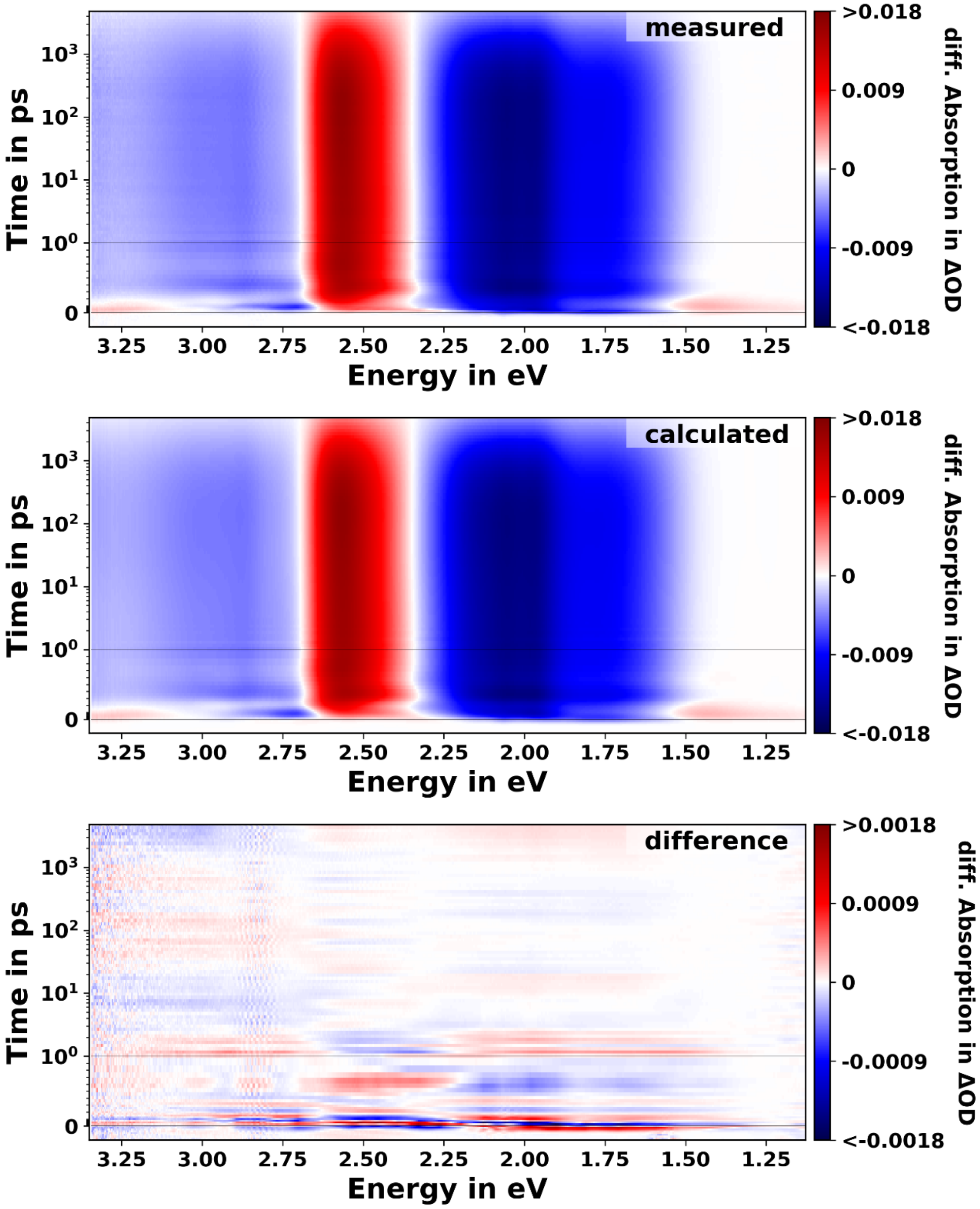

Figure S19. Measured, modelled, and 10x the difference shown as two-dimensional matrices. Fitting with oscillatory temporal parameters fixed, but spectra allowed to vary as described in step 9 in the procedure above using a cosine function for modelling the oscillations.

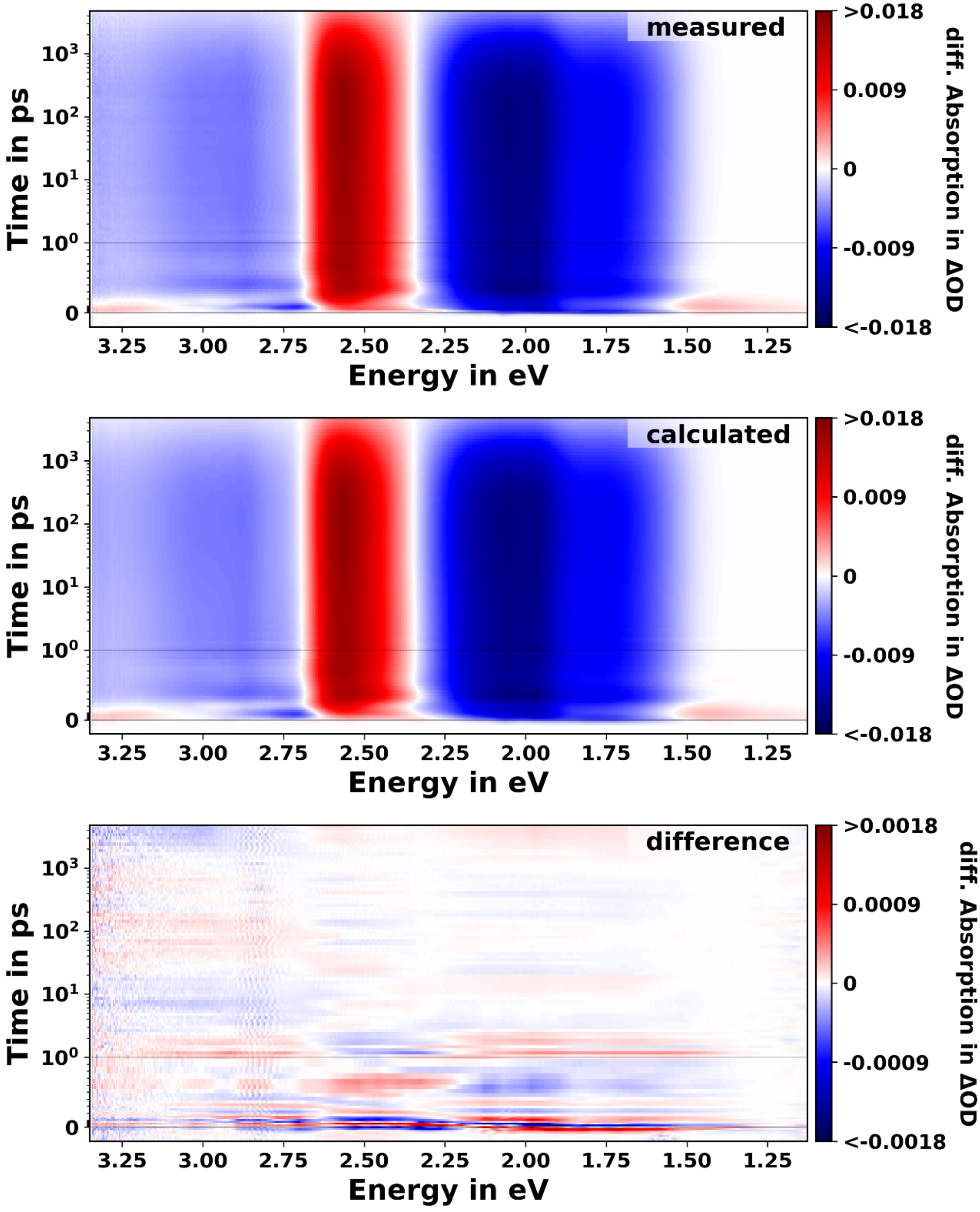

Figure S20. Measured, modelled, and 10x the difference shown as two-dimensional matrices. Fitting with all parameters allowed to vary as described in step 10 in the procedure above using a cosine function for modelling the oscillations.

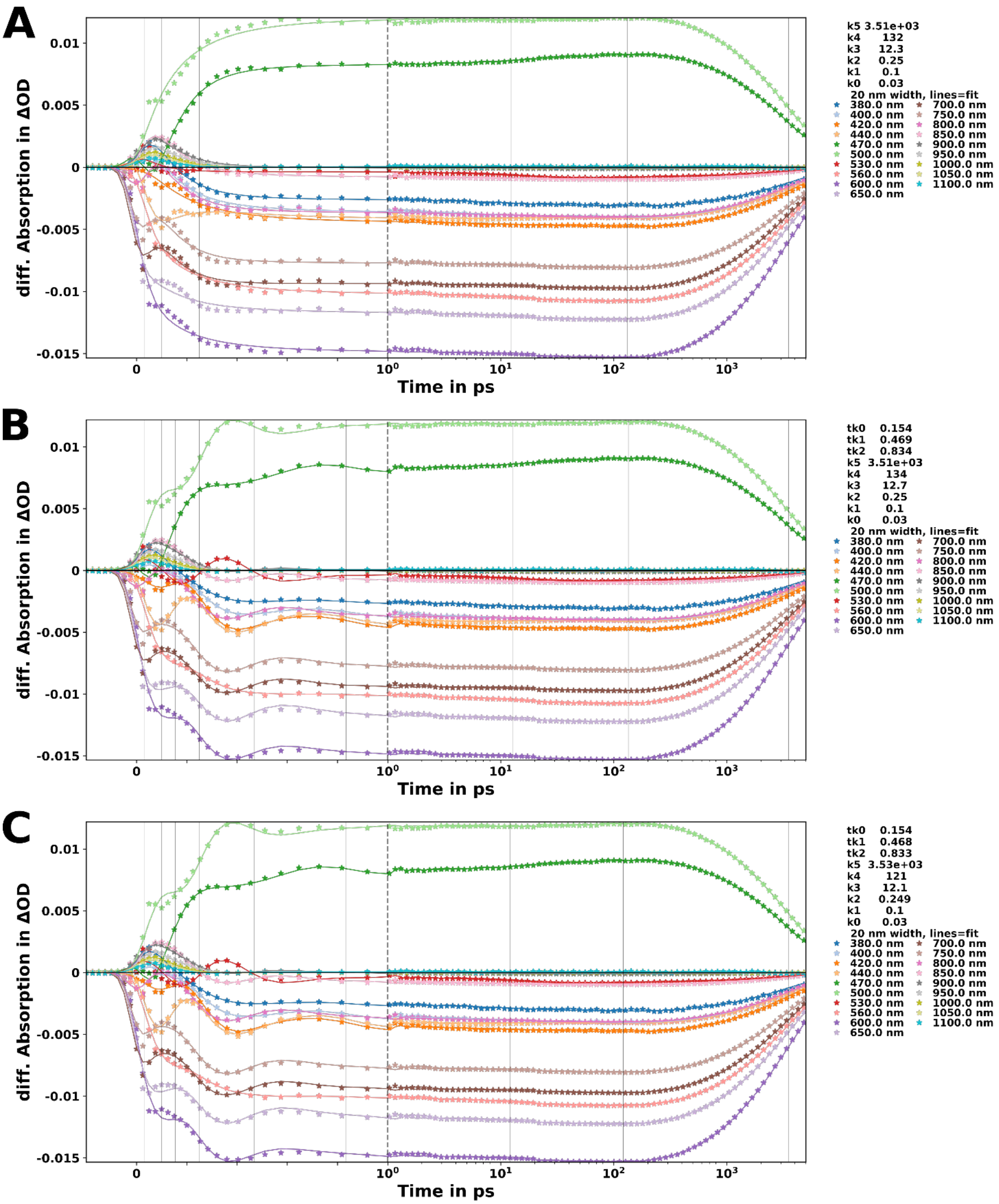

Figure S21. Kinetic development of the data for $(^{CF3}L^1)_2Fe$. Dots are measured and chirp corrected datapoints. The lines originate from (a) Step 8 in the description above; (b) and (c) Steps 9 and 10 in the description above. In this model, the oscillations were modeled using a cosine function.

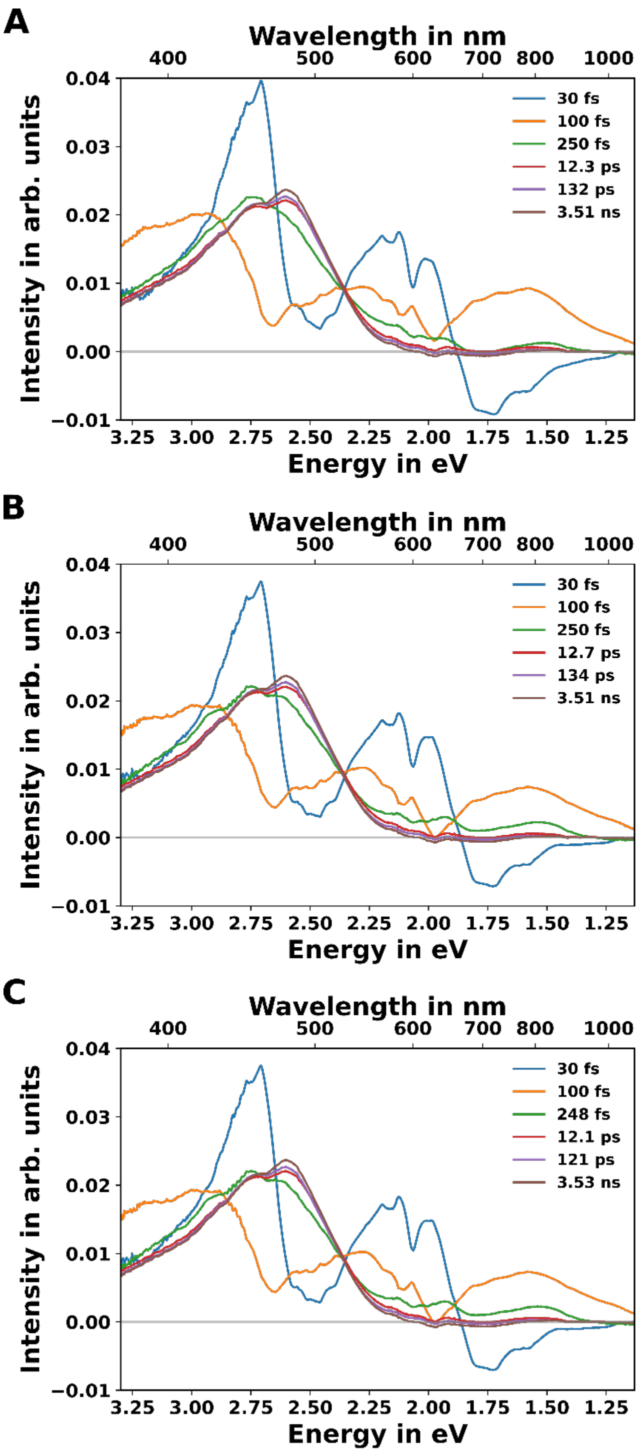

Figure S22. Extracted species-associated spectra from the fits for $(^{CF3}L^{1})_2Fe$ using a cosine function. (a) Kinetic modelling in Step 8 with removed oscillations and least-square optimizer. (b) Kinetic and spectral modelling in step 9 with locked oscillation temporal parameter. (c) Freely refining temporal and oscillator parameters. From the extracted SAS spectra, the ground state measured absorption spectra were subtracted, after it was scaled to the average intensity of the 10-15 ps feature between 600 and 700nm.

The kinetic parameters are labelled k1 through k6 and have each an initial value, refined value and an upper and lower limit determined by a t-testing of the complete parameter set using the same optimizer. We note that the error values as obtained by the algorithm are extremely narrow, ~5%. When testing the error estimation procedure with other algorithms such as simplex or other (known and constructed) data, the algorithm performs as expected and usually produces significantly wider error margins. We can only assume that the complexity of the model results in a strongly defined local minima.

For more details see the description in the publication associated with the KiMoPack software package and its manual.[5] As given in the tables (Table S1 – Table S9), the rates correspond to the following processes: k1 reflects ^{1}PALCT→^{3}PALCT ISC; k2 corresponds to conversion of ^{3}PALCT into the MC manifold; k3 represents ISC within MC manifold namely ^{3}MC→^{5}MC transition; k6 is the rate of conversion of the ^{5}MC back to ground state; k4 and k5 reflect relaxation processes within the ^{5}MC state. We note the time of ^{1}PALCT→^{3}PALCT ISC is not very well defined as it is shorter than the IRF. The parameters f0-f2 and tk0-tk2 represent the oscillation periods and the dephasing times (that is, the time in which the amplitude of oscillations decay).

All three slow components have very similar SAS which are also very similar to slow ESA extracted in oTA by subtraction of GSB; only subtle differences are evident, reflecting some narrowing of the ESA of what we assign to be the ^{5}MC state. The SAS of the 30 fs component gives a substantially different shape than the SAS of the 100 fs component and to some extent mirrored it, with both fast components in turn looking very different from the SAS of the slower components. We note that in both extractions, an isosbestic point is visible at 530 nm – as also observed in the measured data. The shape of the fastest SAS is reasonable, but the amplitudes of different spectral bands look quite different from the GSB subtraction-generated spectrum. The

difference is somewhat expected as (a) the data-generated spectrum for the component that is faster than the IRF has contributions of slower components, and (b) in the global analysis, the amplitude and the shape of a component faster than IRF may be highly distorted. When we compare SAS of the two earliest components for both fitting with cos(ωt) and sin(ωt) functions we observe that these two components anti-correlate with each other suggesting some sort of compensation effect in the fitting.

Spectral Shapes of Oscillation Amplitudes

Figure S23 shows the expanded comparison of the temporal and spectral components of the oscillations.

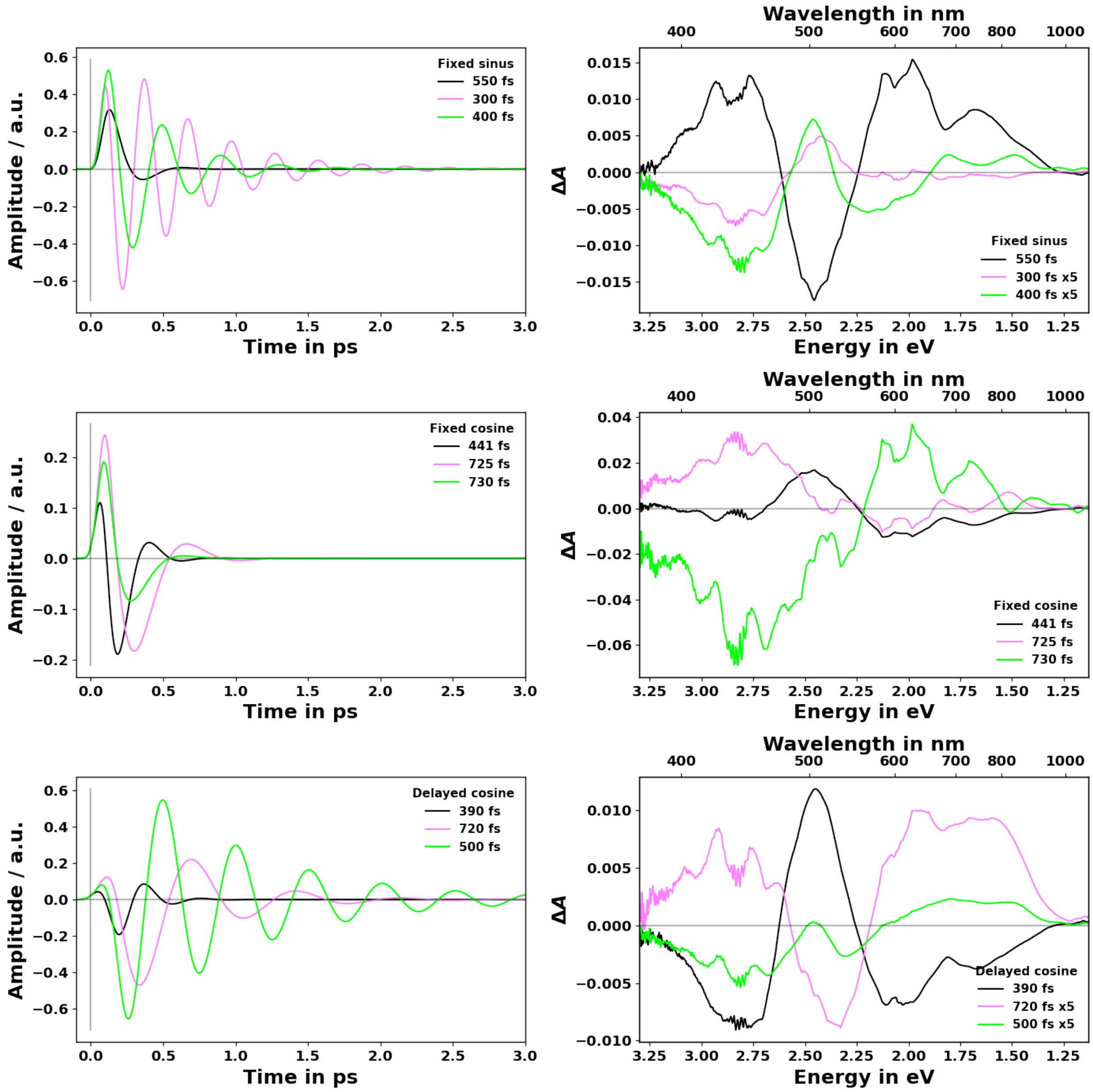

Figure S23. Temporal evolution of the oscillations (left column) and spectra assigned to each oscillation (right column). The selected models are named as title in the legend. Note that some of the amplitudes were amplified, as noted in the respective legends.

In the main text, we refer to comparison of amplitude spectra of oscillations with the ground-state absorption and its derivative, as compared in the figures below:

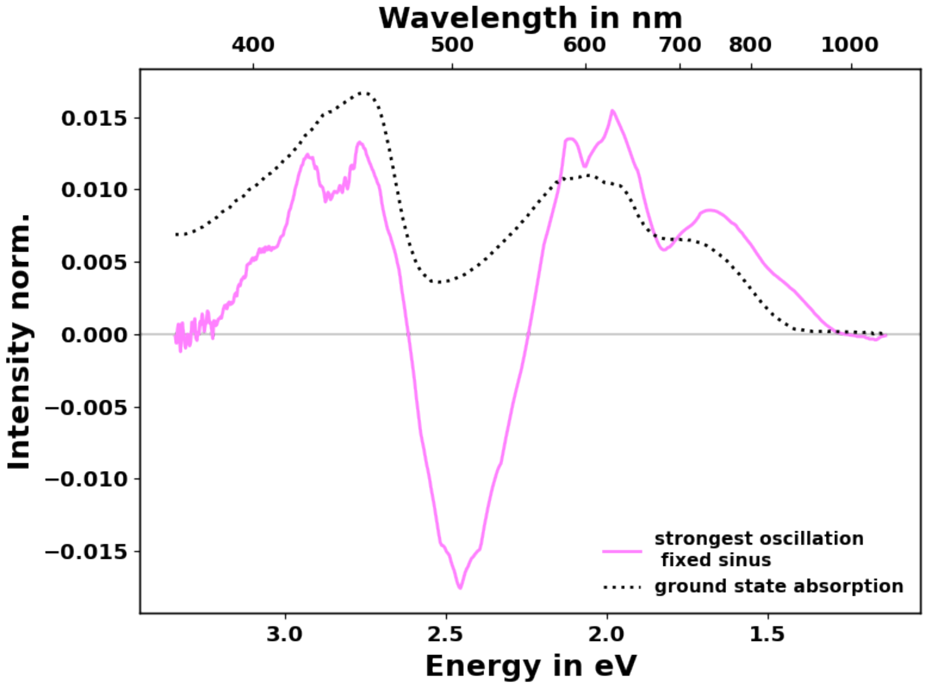

Figure S24. Comparison of the amplitude spectrum of the oscillations with 545 fs period and scaled ground-state absorption spectrum.

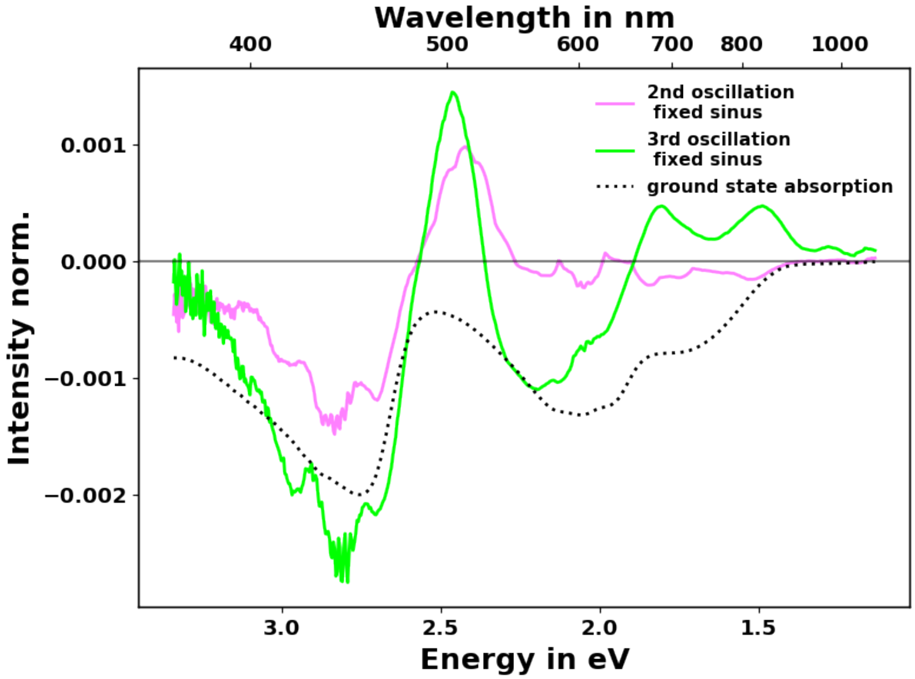

Figure S25. Comparison of the amplitude spectra of oscillations with periods 400 and 300 fs with scaled ground-state absorption and smoothed and scaled derivative of the absorption spectrum.

Ground State Recovery

Also, as stated in the main text, we do not observe any significant signatures of GSR faster than on the ns timescale. We use the following logic to argue on neglecting GSR during excited state-to-state transformation. In general, GSR occurs as a synchronous decay of the ground state bleach (GSB) in the entire spectral region of the ground state absorption. First, let's assume that GSR efficiently competes with early state-to-state transitions. Then the concentration of absorbed excitation photons is (much) larger than the concentration of ^{5}MC state. We know that ^{5}MC state does not undergo any fast GSR as it converts to the ground state (GSR) on 3.6 ns time scale. When we extract ESA of the ^{5}MC state, we scale the inverted ground state absorption to obtain GSB spectrum for subtraction from the TA spectra. In this procedure, in the constructed ^{5}MC ESA we aim at absence of any remaining negative GSB signal as well as of a positive signal with the features of the ground state absorption. As a bonus of this procedure, we identify the concentration of excited molecules at that delays as we know the ground state absorption extinction (molar attenuation coefficient). The same GSB spectrum was used to get ^{3}MC ESA meaning that the concentration of ^{3}MC and ^{5}MC is the same and that the GSR from ^{3}MC is negligible. The very fast ^{1}PALCT state depopulation (due to ISC) leads to the kinetics of ^{1}PALCT state which are close to the IRF and to observation of the largest signal at the time close to zero delay time that corresponds to half of absorbed photons concentration.

To successfully subtract GSB from the ^{1}PALCT state TA spectrum, we scaled down the ^{5}MC state concentration two times which implies that there is no GSR from ^{1}PALCT state. If GSR would be a significant part of the very fast dynamics, maximum concentration of the ^{1}PALCT state should be significantly larger than the ^{5}MC state concentration. Therefore, in the constructed ESA we would need more than half of the amplitude of the GSB to remove the negative TA signal over

the entire spectral range of the ground state absorption, which is not the case in our data analysis. Now, by exclusion, GSR from ^{3}PALCT state should also be weak. Furthermore, to expect a scalable GSR from ^{3}PALCT state we must assume that its couplings to the ground state is much larger than that of the ^{1}PALCT state as its measured depopulation times is only a few times longer than for ^{1}PALCT. This requirement conflicts the expected decrease of the ^{3}PALCT state coupling to the ground state due to the spin parity by two- to four- orders of magnitude, which is an additional argument to neglect GSR from the ^{3}PALCT state. Also, when we remove oscillations from the TA spectral dynamics during fitting, we do not observe any sign of GSR in kinetics (see Figure S26.

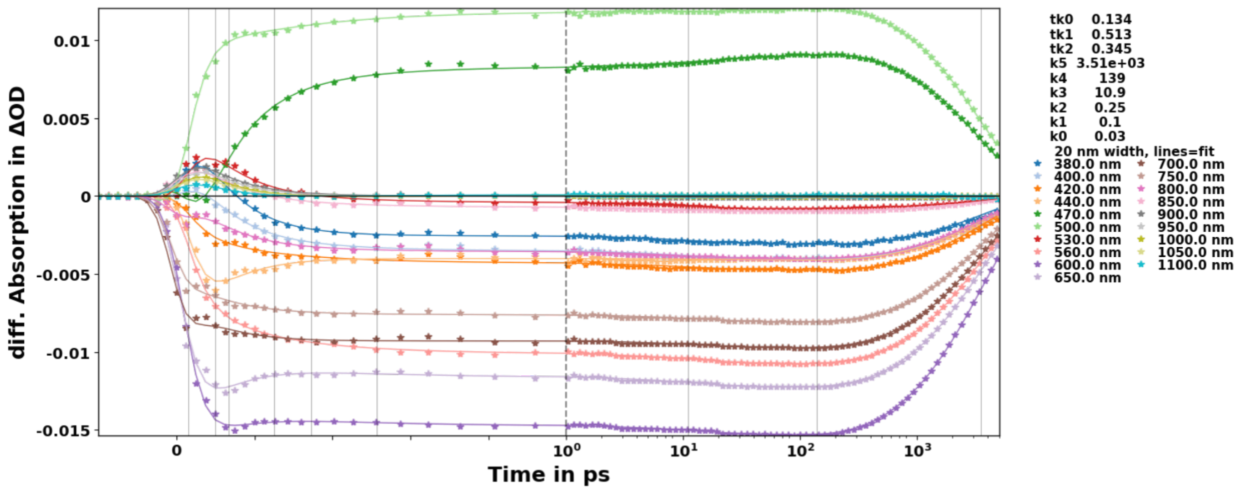

Figure S26. Kinetic development of the data for $(^{CF3}\mathbf{L}^1)_2Fe$ with the fitted oscillations removed from both the data and the fit. This is essentially the same data as in Figure S11c, but now shows only the kinetic evolution of the data.

COMPUTATIONAL FIGURES AND SUPPORTING DISCUSSION

All computations were carried out using Orca v5.0.4.[8–10] Using the previously reported optimized geometry of (**^{CF3}L^1**)$_2$Fe, TD-DFT calculations were performed following the same computational strategy as reported before[1] to generate the electron and hole density distribution map for the vertical transition at 2.05 eV, corresponding to the excitation wavelength of 600 nm used for all transient absorption experiments in this study. Then using the previously reported coordinates of the optimized geometries of the triplet and metal-centered states of a closely related analogue (**^{Cl}L^1**)$_2$Fe,[6] TD-DFT calculations were performed for the ^{3}MC and ^{5}MC states using the B3LYP functional,[11–13] ZORA approximation,[14] and ZORA-def2-TZVP basis set[15] to account for relativistic effects. This level of theory was chosen to simulate the absorption spectra of the ^{3}MC and ^{5}MC states based on our previous work where testing of different functionals for TD-DFT calculations on quintet metal-centered states revealed B3LYP/ZORA-def2-TZVP to match the experimental data the best. We used the resolution of identity approximation (RIJCOSX)[16] to speed up the calculations, the SMD[17] solvation model (toluene), the SARC/J auxiliary basis set,[18–22] and Grimme's D4 dispersion correction.[23,24] The XY data to plot the calculated spectra were generated using Multiwfn version 3.7 using a 0.37 eV broadening. In order to visualize the electron hole density distribution maps, molden files were generated using the orca_2mkl module, and were fed into Multiwfn 3.7[25] to generate distribution maps at an isosurface value of 0.002. The optimized geometries were visualized using Mercury.[26] The optical characterization of (**^{Cl}L^1**)$_2$Fe , which is nearly identical to that of (**^{CF3}L^1**)$_2$Fe, will be reported in a subsequent paper. We include here selections from its computational analysis that are important for analysis of the dynamic behaviour of (**^{CF3}L^1**)$_2$Fe.

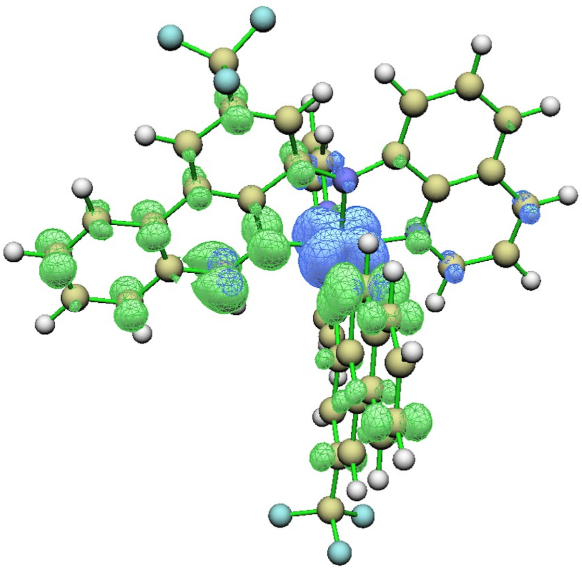

Figure S27. Electron (green) and hole (blue) density distribution maps (isosurface = 0.002) for the 2.05 eV (~600 nm) transition of $(^{CF3}\mathbf{L}^{1})_2Fe$.

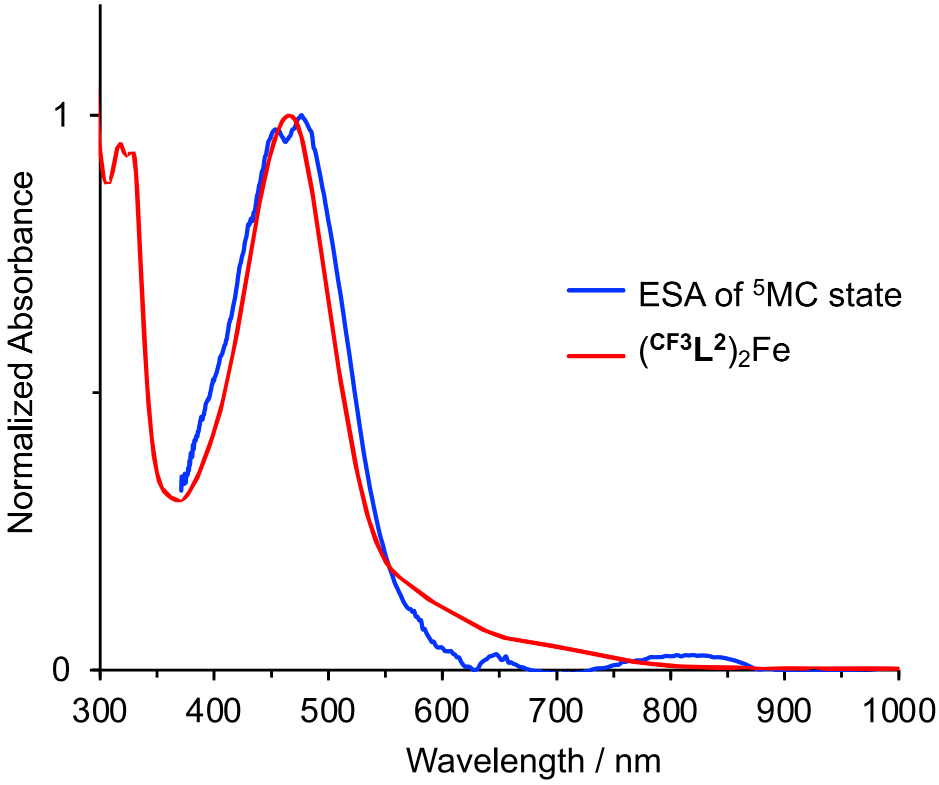

Figure S28. Overlay of the ESA attributed to the ^{5}MC state (2 ps) and the experimental absorption spectrum of the high-spin model complex $(^{CF3}\mathbf{L}^{2})_2Fe$.

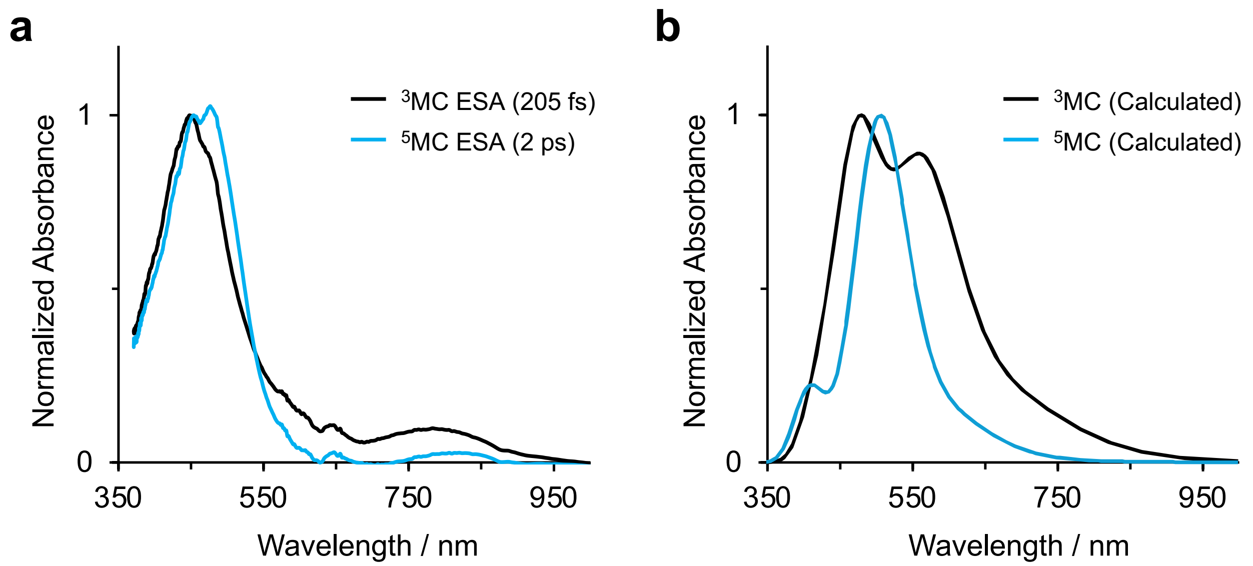

Figure S29. Comparison of (a) the ESA spectra attributed to the ^{3}MC state (205 fs) and ^{5}MC state (2 ps) of ($^{\mathbf{CF3}}\mathbf{L^1}$)$_2$Fe (b) TD-DFT simulations in toluene (SMD-D4-B3LYP-RIJCOSX-ZORA/ZORA-def2-TZVP+SARC/J; FWHM = 0.37 eV) of the absorption spectra using the optimized geometries of the ^{3}MC and ^{5}MC excited states of the closely related analogue ($^{\mathbf{Cl}}\mathbf{L^1}$)$_2$Fe.

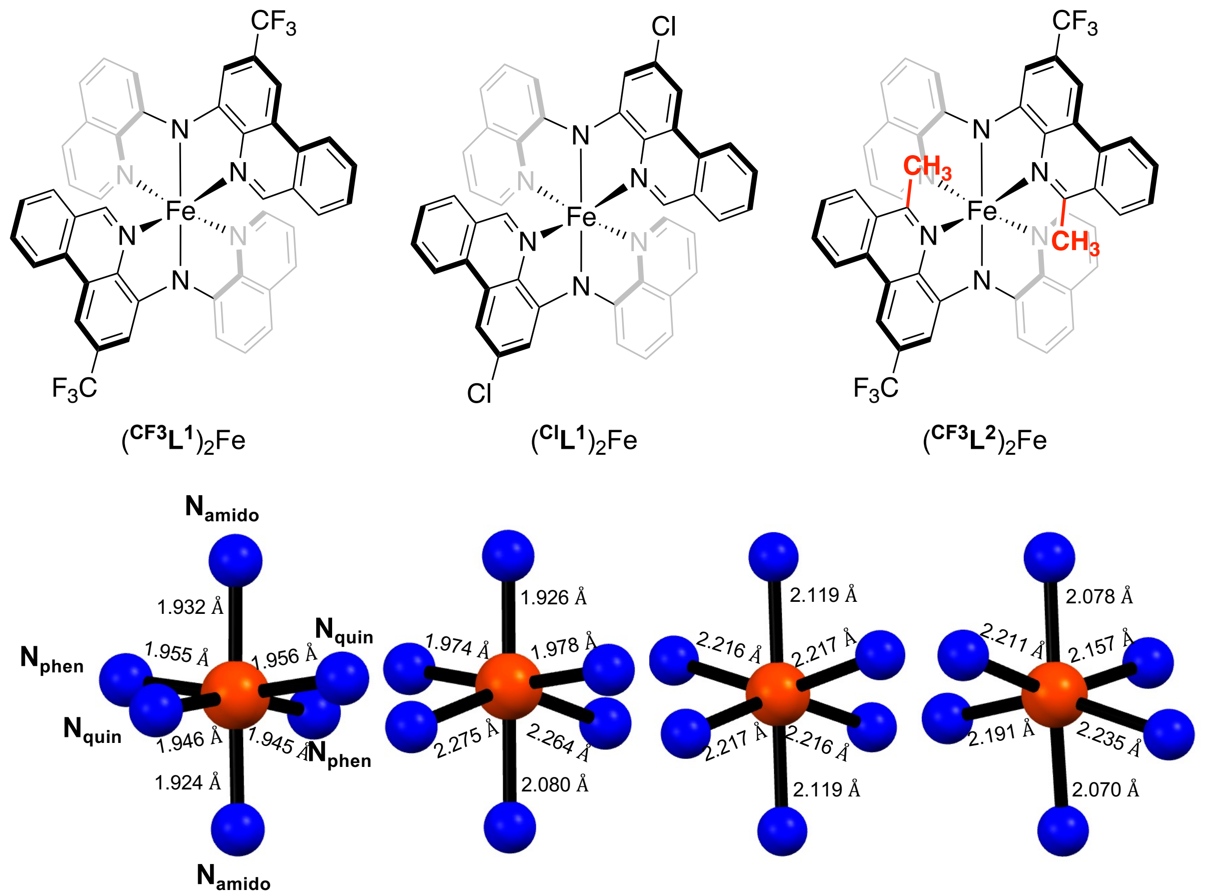

Parameter	$(^{CF3}L^1)_2Fe$ (XRD)	^3MC $(^{Cl}L^1)_2Fe$ (DFT)	^5MC $(^{Cl}L^1)_2Fe$ (DFT)	$(^{CF3}L^2)_2Fe$ (XRD)
d_{mean}^{a} / Å	1.943	2.083	2.184	2.157
ζ^b / Å	0.0607	0.746	0.261	0.332
Δ^c	0.00004	0.00450	0.00045	0.00086
Σ^d / °	58.86	95.04	132.14	123.55
Θ^e / °	195.17	339.61	410.32	441.08
Volume / Å^3	9.64	11.49	12.99	12.45

^{a} d_{mean} = average metal-ligand bond length; ^{b} $\zeta = \sum_{i=1}^{6}|d_i - d_{mean}|$; ^{c} $\Delta = \frac{1}{6}\sum_{i=1}^{6}\left(\frac{d_i - d_{mean}}{d_{mean}}\right)^2$; ^{d} $\Sigma = \sum_{i=1}^{12}|\phi_i - 90|$;

^{e} $\Theta = \sum_{i=1}^{24}|\theta_i - 60|$

Figure S30. Calculated octahedricity parameters[27] for the optimized geometries of the ^{3}MC and ^{5}MC excited states of $(^{Cl}\mathbf{L}^1)_2$Fe, and comparison with the ground-state of $(^{CF3}\mathbf{L}^1)_2$Fe and the high-spin model complex $(^{CF3}\mathbf{L}^2)_2$Fe.

REFERENCES

(1) Braun, J. D.; Lozada, I. B.; Kolodziej, C.; Burda, C.; Newman, K. M. E.; Van Lierop, J.; Davis, R. L.; Herbert, D. E. Iron(Ii) Coordination Complexes with Panchromatic Absorption and Nanosecond Charge-Transfer Excited State Lifetimes. *Nat. Chem.* **2019**, *11*, 1144–1150.

(2) Reinhard, M. E.; Sidhu, B. K.; Lozada, I. B.; Powers-Riggs, N.; Ortiz, R. J.; Lim, H.; Nickel, R.; Lierop, J. van; Alonso-Mori, R.; Chollet, M.; Gee, L. B.; Kramer, P. L.; Kroll, T.; Raj, S. L.; van Driel, T. B.; Cordones, A. A.; Sokaras, D.; Herbert, D. E.; Gaffney, K. J. Time-Resolved X-Ray Emission Spectroscopy and Synthetic High-Spin Model Complexes Resolve Ambiguities in Excited-State Assignments of Transition-Metal Chromophores: A Case Study of Fe-Amido Complexes. *J. Am. Chem. Soc.* **2024**, *146*, 17908–17916.

(3) Fulmer, G. R.; Miller, A. J. M.; Sherden, N. H.; Gottlieb, H. E.; Nudelman, A.; Stoltz, B. M.; Bercaw, J. E.; Goldberg, K. I. NMR Chemical Shifts of Trace Impurities: Common Laboratory Solvents, Organics, and Gases in Deuterated Solvents Relevant to the Organometallic Chemist. *Organometallics* **2010**, *29*, 2176–2179.

(4) Krejčik, M.; Daněk, M.; Hartl, F. Simple Construction of an Infrared Optically Transparent Thin-Layer Electrochemical Cell: Applications to the Redox Reactions of Ferrocene, Mn2(CO)10 and Mn(CO)3(3,5-Di-t-Butyl-Catecholate)−. *J. Electroanal. Chem. Interf. Electrochem.* **1991**, *317*, 179–187.

(5) Müller, C.; Pascher, T.; Eriksson, A.; Chabera, P.; Uhlig, J. KiMoPack: A Python Package for Kinetic Modeling of the Chemical Mechanism. *J. Phys. Chem. A* **2022**, *126*, 4087–4099.

(6) Larsen, C. B.; Braun, J. D.; Lozada, I. B.; Kunnus, K.; Biasin, E.; Kolodziej, C.; Burda, C.; Cordones, A. A.; Gaffney, K. J.; Herbert, D. E. Reduction of Electron Repulsion in Highly Covalent Fe-Amido Complexes Counteracts the Impact of a Weak Ligand Field on Excited-State Ordering. *J. Am. Chem. Soc.* **2021**, *143*, 20645–20656.

(7) Gavana, A. *Python implementation of Ampgo*. http://infinity77.net/global_optimization/index.html.

(8) Neese, F. The ORCA Program System. *WIREs Comput. Mol. Sci.* **2012**, *2*, 73–78.

(9) Neese, F.; Wennmohs, F.; Becker, U.; Riplinger, C. The ORCA Quantum Chemistry Program Package. *J. Chem. Phys.* **2020**, *152*, 224108.

(10) Neese, F. Software Update: The ORCA Program System—Version 5.0. *WIREs Comput. Mol. Sci.* **2022**, *12*, e1606.

(11) Lee, C.; Yang, W.; Parr, R. G. Development of the Colle-Salvetti Correlation-Energy Formula into a Functional of the Electron Density. *Phys. Rev. B* **1988**, *37*, 785–789.

(12) Becke, A. D. A New Mixing of Hartree–Fock and Local Density-Functional Theories. *J. Chem. Phys.* **1993**, *98*, 1372–1377.

(13) Becke, A. D. Density-Functional Thermochemistry. III. The Role of Exact Exchange. *J. Chem. Phys.* **1993**, *98*, 5648–5652.

(14) Van Wüllen, C. Molecular Density Functional Calculations in the Regular Relativistic Approximation: Method, Application to Coinage Metal Diatomics, Hydrides, Fluorides and Chlorides, and Comparison with First-Order Relativistic Calculations. *J. Chem. Phys.* **1998**, *109*, 392–399.

(15) Weigend, F.; Ahlrichs, R. Balanced Basis Sets of Split Valence, Triple Zeta Valence and Quadruple Zeta Valence Quality for H to Rn: Design and Assessment of Accuracy. *Phys. Chem. Chem. Phys.* **2005**, *7*, 3297–3305.

(16) Neese, F.; Wennmohs, F.; Hansen, A.; Becker, U. Efficient, Approximate and Parallel Hartree–Fock and Hybrid DFT Calculations. A 'Chain-of-Spheres' Algorithm for the Hartree–Fock Exchange. *Chem. Phys.* **2009**, *356*, 98–109.

(17) Marenich, A. V.; Cramer, C. J.; Truhlar, D. G. Universal Solvation Model Based on Solute Electron Density and on a Continuum Model of the Solvent Defined by the Bulk Dielectric Constant and Atomic Surface Tensions. *J. Phys. Chem. B* **2009**, *113*, 6378–6396.

(18) Weigend, F. Accurate Coulomb-Fitting Basis Sets for H to Rn. *Phys. Chem. Chem. Phys.* **2006**, *8*, 1057–1065.

(19) Pantazis, D. A.; Chen, X.-Y.; Landis, C. R.; Neese, F. All-Electron Scalar Relativistic Basis Sets for Third-Row Transition Metal Atoms. *J. Chem. Theory Comput.* **2008**, *4*, 908–919.

(20) Pantazis, D. A.; Neese, F. All-Electron Scalar Relativistic Basis Sets for the Lanthanides. *J. Chem. Theory Comput.* **2009**, *5*, 2229–2238.

(21) Pantazis, D. A.; Neese, F. All-Electron Scalar Relativistic Basis Sets for the 6p Elements. *Theor Chem Acc* **2012**, *131*, 1–7.

(22) Pantazis, D. A.; Neese, F. All-Electron Scalar Relativistic Basis Sets for the Actinides. *J. Chem. Theory Comput.* **2011**, *7*, 677–684.

(23) Caldeweyher, E.; Bannwarth, C.; Grimme, S. Extension of the D3 Dispersion Coefficient Model. *J. Chem. Phys.* **2017**, *147*, 034112.

(24) Caldeweyher, E.; Ehlert, S.; Hansen, A.; Neugebauer, H.; Spicher, S.; Bannwarth, C.; Grimme, S. A Generally Applicable Atomic-Charge Dependent London Dispersion Correction. *J. Chem. Phys.* **2019**, *150*, 154122.

(25) Lu, T.; Chen, F. Multiwfn: A Multifunctional Wavefunction Analyzer. *J. Comput. Chem.* **2012**, *33*, 580–592.

(26) Macrae, C. F.; Sovago, I.; Cottrell, S. J.; Galek, P. T. A.; McCabe, P.; Pidcock, E.; Platings, M.; Shields, G. P.; Stevens, J. S.; Towler, M.; Wood, P. A. Mercury 4.0: From Visualization to Analysis, Design and Prediction. *J. Appl. Cryst.* **2020**, *53*, 226–235.

(27) Ketkaew, R.; Tantirungrotechai, Y.; Harding, P.; Chastanet, G.; Guionneau, P.; Marchivie, M.; Harding, D. J. OctaDist: A Tool for Calculating Distortion Parameters in Spin Crossover and Coordination Complexes. *Dalton Trans.* **2021**, *50*, 1086–1096.
